# Language Usage and Second Language Morphosyntax: Effects of Availability, Reliability, and Formulaicity

**DOI:** 10.3389/fpsyg.2021.582259

**Published:** 2021-04-29

**Authors:** Rundi Guo, Nick C. Ellis

**Affiliations:** Language Learning Laboratory, Department of Psychology, University of Michigan, Ann Arbor, MI, United States

**Keywords:** SLA, morphosyntax, availability, reliability, formulaicity, phrase-superiority effects, elicited imitation task

## Abstract

A large body of psycholinguistic research demonstrates that both language processing and language acquisition are sensitive to the distributions of linguistic constructions in usage. Here we investigate how statistical distributions at different linguistic levels – morphological and lexical (Experiments 1 and 2), and phrasal (Experiment 2) – contribute to the ease with which morphosyntax is processed and produced by second language learners. We analyze Chinese ESL learners’ knowledge of four English inflectional morphemes: *-ed*, *-ing*, and third-person *-s* on verbs, and plural *-s* on nouns. In Elicited Imitation Tasks, participants listened to length- and difficulty-matched sentences each containing one target morpheme and typed the whole sentence as accurately as they could after a short delay. Experiment 1 investigated lexical and morphemic levels, testing the hypotheses that a morpheme is expected to be more easily processed when it is (1) highly *available* (i.e., occurring in frequent word-forms), and (2) highly *reliable* (i.e., occurring in lemma words that are consistently conjugated in the form containing this morpheme). Thirty sentences were made for each morpheme, divided into three Availability-Reliability Distribution (ARD) groups on the basis of corpus analysis in the Corpus of Contemporary American English (COCA; Davies, 2008-): 10 target words high in availability, 10 high in reliability, and 10 low in both reliability and availability. Responses were scored on whether the target morpheme was accurately reproduced given the provision of the correct lemma. A generalized linear mixed-effects logit model (GLMM) revealed fixed effects of morpheme type, availability, and reliability on the accuracy of morpheme provision. There were no effects of lemma frequency. Experiment 2 successfully replicated these results and extended the investigation to explore phrasal formulaicity by manipulating the frequency of the four-word strings in which the morpheme was embedded. GLMMs replicated the effects of word-form availability and reliability and additionally revealed independent *phrase-superiority effects* where morphemes were better reproduced in contexts of higher string-frequency. Taken together, these findings demonstrate that morpheme acquisition reflects the distributional properties of learners’ experience and the mappings therein between lexis, morphology, phraseology, and semantics. These conclusions support an emergentist view of the statistical symbolic learning of morphology where language acquisition involves the satisfaction of competing constraints across multiple grain*-*sizes of units.

## Introduction

Novice and intermediate learners of English as a second language (ESL) are far from consistent in their production of inflectional morphemes, such as regular past-tense *-ed*, or third person singular present-tense *-s*. Jia and Fuse (2007) show that the acquisition of a morpheme such as the third-person singular *-s* can take 5 years or more to go from 0 to 80% provision in obligatory contexts for ESL children. Five years of English usage involves many thousands of receptive experiences of high frequency functional morphemes, and many thousands of contexts requiring their productive use, yet provision is variable. This suggests that the system is learned incrementally, and that regularities/generalization/productivity emerge from the combined experience of usage. But is it the case that, for any given morpheme, some exemplars are more easily recognized in the input and produced earlier in acquisition? If so, what are these exemplars that are more likely to be preferentially processed? And why these bellwethers? Are they special in their distributional statistics, for example, in terms of their frequency, or their form-function contingency, or their formulaicity? These are the questions that motivate our research here. How does second language (L2) morphological ability depend upon usage?

Usage-based theories hold that domain-general cognitive mechanisms drive the learning of linguistic constructions and the emergence of generalizations (e.g., Goldberg, 2006; Beckner et al., 2009; MacWhinney and O’Grady, 2014; Wulff and Ellis, 2018). They proposed that acquisition is modulated by factors affecting attention and memory, such as exemplar type- and token-frequency, contingency of form-function mapping, salience of form and of function, paradigm complexity, neighborhood effects and the proportion of friends to enemies^1^ in quasi-regular domains, etc. (e.g., Marchman, 1997; MacWhinney, 2001; Bybee, 2006; Ellis, 2006a, b; Seidenberg and Plaut, 2014). For the case of morphology, we might ask then, in the 5 years during which L2 learners are learning to produce third-person singular *-s*, do experiences of particular *-s* inflected verbs play a role in the acquisition of the system more than others? Likewise, for the even more extended period during which L2 learners are learning to produce regular past-tense *-ed*, are particular *-ed* inflected verbs more potent exemplars than others? And so on. From studies of children (Brown, 1973; Braine et al., 1990; Ambridge et al., 2015; Finley, 2018) and of adults (e.g., Seidenberg and Plaut, 2014; Pollatsek et al., 2015), there is good reason to suspect that distributional factors affect L1 and L2 morpheme acquisition and processing.

Linguistic constructions vary in frequency and they distribute across usage in complex probabilistic patterns. Psycholinguistics research has established several important aspects of these distribution patterns. The most studied parameter is *availability*, which concerns how often a language learner experiences a given form in their usage history. Availability is estimated as the normalized token frequency of a specific word-form in representative corpora. For example, the availability of the word-form *depends* is the overall probability of encountering the word-form *depends* in English usage, i.e., P(*depends*). The effects of availability on the development and processing of L1 and L2 has been well-established. For example, words high in frequency are named faster (Forster and Chambers, 1973; Seidenberg and McClelland, 1989), judged faster in lexical decision tasks (Yap and Balota, 2014), fixated for shorter durations in reading (McDonald and Shillcock, 2003), recognized more easily in speech (Luce, 1986), and spelled more accurately (Barry and Seymour, 1988). More generally, language learners are sensitive to the frequency of linguistic cues across a wide range of linguistic domains and levels of representation, including phonology and phonotactics, lexis, reading and spelling, morphosyntax, sentence comprehension, etc. (Bybee and Hopper, 2001; Ellis, 2002; Bod et al., 2003).

For the particular case of morphology, words inflected in a form that is high in token frequency are produced earlier and more accurately in that form compared to in other forms and compared to other words that are inflected in low token frequency forms. Such token frequency effects of word-forms have been reported in the acquisition of L1 (Marchman, 1997; Ambridge et al., 2015; Räsänen et al., 2016), L2 (Larsen-Freeman, 1976; Goldschneider and DeKeyser, 2001; Jia and Fuse, 2007), and artificial grammars (Braine et al., 1990; Finley, 2015). Notably, the frequency of word lemmas plays a lesser role in the accurate retrieval of inflected word-forms as compared to the token frequency of the inflected word-form itself - a key finding that has important implications for emergentist approaches which posit chunk-based learning from usage, construction grammar, and linguistic structure as processing history.

Another important distribution parameter is *reliability*, i.e., how likely it is that a linguistic cue reliably co-occurs either with another construction, or with a particular interpretation. Measuring reliability entails the statistical estimation of some form of contingency (MacWhinney, 2001; Ellis, 2006a; Gries and Ellis, 2015). In the context of morpheme acquisition, reliability can be understood as the relative frequency of different word-forms of a lemma, for example, the reliability of the lemma [depend] occurring in its *-s* morpheme inflected form *depends* can be calculated as the number of occurrences of the word-form *depends* divided by the number of occurrences of all possible word-forms of the lemma [depend] such as *depend*, *depending*, and *depended*, i.e., P (*depends*| [depend]). To the native ear, *depends* might well sound more natural than *depended*, perhaps due to the fact that in an English-speaking environment, when the word *depend* is used, it is most often conjugated in its third-person singular form. In other words, the high reliability of *depends* might facilitate its processing in this form, regardless of the overall frequency of occurrence of *depends* in the entire environment. As a result, *depend* might become implicitly more associated with the morpheme *-s* and with the present than with the other tenses. Psychological research into animal and human learning alike demonstrates profound and ubiquitous impacts of contingency in the learning of cue-outcome associations (Shanks, 1995).

The relative frequency of different morphological forms of the same word have been found to predict usage, language change, accuracy, and error patterns in language processing and acquisition (Bybee, 1985; Hay, 2001; Matthews and Theakston, 2006; Sugaya and Shirai, 2009; Tatsumi et al., 2018). Hay’s (2001) study of relative frequency in derivational morphology, which follows proposals on the structure of paradigms (groups of inflectionally related words with a common lexical stem) in Bybee (1985, Chapter 3), demonstrates that the more frequent member of a paradigm is more accessible and less compositional. Paradigms consist of words of different frequency/accessibility levels, the high frequency words are dominant, and the others are dependent upon them. In studies of language change, paradigms are more likely to be re-made on the basis of the highest frequency form (Bybee, 1985). In studies of L1 acquisition, when acquiring irregular plural forms, English speaking children tend to erroneously produce phrases like *^∗^two mouse* much more frequently than phrases like *^∗^two tooth*, likely because *mouse* is a much more reliable form for the lemma [mouse] than *tooth* is for [tooth]: the word-form *mouse* occurs seven times more than the word-form *mice*, whereas the word-form *tooth* occurs only one sixth as often as does *teeth* (Matthews and Theakston, 2006).

The third distribution parameter to be considered here is *formulaicity*, i.e., the frequency of the multi-word strings in which a morpheme-inflected word-form is embedded^2^. Consider how you might more naturally end the phrase *“you’ve got to be …”* with *“kidding”* than with *“playing.”* According to the Corpus of Contemporary American English (Davies, 2008-), the multi-word string *“you’ve got to be kidding”* is more frequently used than *“you’ve got to be playing”*; i.e., P *(“you’ve got to be kidding”*) > P (*“you’ve got to be playing”*). Note that this is an effect of string frequency, since P (*“kidding”*) < P (*“playing”*).

High-frequency phrases, idioms, and formulaic sequences (Sinclair, 1995; Wray, 2002) are processed more fluently than matched low-frequency strings. For example, in phrasal decision tasks (Bod, 2001; Jiang and Nekrasova, 2007; Arnon and Snider, 2010), high frequency phrasal constituents or short sentences (e.g., *don’t have to worry*; *I like it*) are judged to be grammatical phrases faster than less frequent controls composed of frequency-matched component words (*don’t have to wait*; *I keep it*). Formulaicity effects have likewise been demonstrated in L1 acquisition by Bannard and Matthews (2008) who showed that 2–3-year-old English speaking children were quicker and more accurate at repeating frequently occurring multi-word strings (e.g., “sit in your chair”) sampled from a large child-directed speech corpus, compared to matched infrequent strings (“sit in your truck”). High-frequency slot-and-frame patterns (Braine, 1976) or frames (Mintz, 2003) can strongly constrain the nature of the slot-filler, e.g., a frame like ‘‘to __ it’’ is highly predictive of verbs^3^. Such distributional information can be potent in the acquisition of both the grammatical and the semantic properties of the slot-filler (Elman, 1990; Redington et al., 1998). Mintz et al. (2014) compared training situations in which target words (such as *lowfa*) occurred surrounded by two-word frames (such as *swetch_klide*) that frequently co-occurred, against situations in which target words occurred in simpler bigram contexts (such as *swetch lowfa* or *lowfa klide*) where only an immediately adjacent word provides the context for categorization). They found that learners categorized words together when they occurred in similar frame contexts, but not when they occurred in similar bigram contexts. In a study of L1 English-speaking 2 1/2-year-olds, Childers and Tomasello (2001) found that a nonce verb was better acquired so to be subsequently used creatively in a transitive utterance when it was surrounded by pronouns than when surrounded by proper nouns or names, suggesting that the child’s transitive schema may start out with pronouns in pre-/post-verbal positions (i.e., pronoun V pronoun) rather than being fully general. In other words, frequent formulaic frames can positively promote the processing and productivity of their subcomponent words.

Together, these studies demonstrate that the three distributional factors of *availability*, *reliability*, and *formulaicity* pervasively affect language acquisition and processing. In the current study, we are concerned with their roles in L2 morphology, and whether particular exemplars are more easily recognized in the input and correctly produced because of their privileged distributions in the language.

We examine L2 knowledge of four common English inflectional morphemes (the regular past-tense ending *-ed*, the progressive marker *-ing*, the third-person singular present-tense ending *-s*, and the nominal plural marker *-s*). We target ESL learners whose native language is Mandarin Chinese because this population has been shown to experience greater challenges in acquiring L2 inflections due to the fact that Mandarin Chinese has minimal verb-tense and noun morphology (Yeh et al., 2015). None of the four English morphemes included in our study has a direct morphological equivalence in Mandarin Chinese, although some of them can be expressed with non-inflectional grammatical cues, e.g., certain classifiers that can express plurality, certain aspectual markers (e.g., V-le) that arguably possess properties of a tense marker (Ross, 1995), and lexical cues such as numbers and adverbs. We aim to assess how much ESL morpheme processing and production depends upon their English usage distributions.

Experiment 1 investigates distributions at lexical and morphological levels. Experiment 2 extends the study to include the effects of the distributions of larger phraseological constructions on the processing of embedded morphemes.

## Experiment 1

Experiment 1 investigates the effects of availability and reliability of the word-forms containing target morphemes on the production accuracy of the target morphemes. We hypothesize that a morpheme is more easily processed (1) when it occurs in a word-form that is highly frequent in usage (i.e., highly available), and (2) when it is attached to a word that is more consistently conjugated in the form containing this morpheme compared to other forms of the same word lemma (i.e., highly reliable).

Many studies of morpheme acquisition, following Brown (1973), assess spontaneous production of target morphemes in obligatory contexts, i.e., where the morpheme would be obligatory in a native-speaking adult’s speech either because of the pragmatic context of discourse (e.g., describing something happened in the past calls for use of past-tense verbs) or the syntactic structure of the utterance (e.g., “Yesterday, I walk__ to the store” requires the regular past-tense morpheme *-ed*). Here, instead, we use an Elicited Imitation Task (EIT) with morphemes in non-obligatory contexts of the sort used by Marchman (1997) who investigated the production of the past-tense -ed morpheme in L1 English-speaking children.

### Elicited Imitation Tasks

Elicited Imitation Tasks have been widely used in studies of L2 processing and have been shown to have high validity and reliability (Ortega et al., 2002; Erlam, 2006; Gaillard and Tremblay, 2016). In one version of EIT, the participant hears a sentence and is asked to repeat the exact sentence after a short delay. Unlike production in uncontrolled spontaneous speech (e.g., Jia and Fuse, 2007), EIT allows controlled examination of morpheme production in contexts that are matched in important respects, thus isolating the effects of the property of the morphemes themselves from that of their context (Erlam, 2006). The use of predetermined sentence stimuli allows the control of important potential confounds such as the presence of an adverbial tense cues, the frequency of the strings of words that contain the morpheme, grammatical complexity, memory load, etc.

For present purposes, we modified the EIT design to require participants to ‘repeat’ the sentence by typing the written form rather than speaking the oral form. This modification circumvents accent-induced transcription ambiguity, facilitates data collection and analysis, and is less threatening to our Chinese ESL participants who reportedly experience considerable discrepancy between their proficiency in spoken and written forms of English as a result of classroom pedagogical practices in China, which commonly deemphasize oral English instruction (Ren, 2011).

### A Process Analysis of EIT as It Relates to Morpheme Production

Each of the 120 randomized trials of our EIT involved listening to a single sentence out of context and then, after a short delay during which the participant rates it for how sensible it is, repeating it verbatim, in all of its parts, as accurately as possible. What processes might be involved in the successful repetition of a target morpheme in such a task? The following sketch is informed by proposals in usage-based linguistics, construction grammar, the psycholinguistics of sentence processing, predictive processing, and first and second language acquisition [see, particularly, Christiansen and Chater (2016) on “Chunk-and-Pass” processing, and, more generally for review of language emergence, MacWhinney and O’Grady, 2014]:

The perception and comprehension encoding stages in EIT involve three parts: (1) taking in word-forms into an auditory/lexical buffer, (2) linking lexical items syntactically, and (3) constructing a meaningful interpretation of the sentence. Based on the psycholinguistic research which has shown a variety of frequency effects in the perception and processing of words, morphemes, multi-word chunks, and syntactic constructions, we propose that the initial recognition and preservation of the correct form of target words is likely influenced by the forces of availability, reliability, and formulaicity in terms of storage in an auditory/lexical buffer. Then, the language system rapidly integrates all available incoming information, interactively satisfying multiple constraints as quickly as possible, to update the current interpretation of what has been said so far. Relevant cues include sentence-internal information about lexical and structural biases, as well as extra-sentential cues from the referential and pragmatic context (although the decontextualized nature of EIT denies many of these usual additional influences). As the incoming auditory information is chunked, it is rapidly integrated with contextual information to recognize words and morphemes, which are in turn chunked into larger multiword units. Incremental identification of incoming units is influenced by the sequential probabilities of what has been processed to date: the next word in a well-entrenched word sequence is more easily identified, as is an incoming morpheme that is highly predicted in its context. In parsing and interpreting the target morphemes, there are potential influences of syntactic integrity, e.g., auxiliary [be] impacting particularly progressive *-ing*, and of contextual support where context could influence the encoding of the past *-ed*. The encoding of third person present *-s* and plural *-s* on subjects should also be under the influence of syntactic integrity, although in English, agreement processing is generally less obligatory than processing for tense and aspect (MacWhinney, 1997, 2001). The final stage of EIT, (4) production, is also expected to be sensitive to frequency effects and sequential probabilities at word, morpheme, and particularly phrasal levels: a well-entrenched formulaic phrase will support provision of its component morphemes whether they are analyzed or not. A relevant process analysis of the imitative written production of a recently heard message might look quite like that for speaking (e.g., Levelt, 1989) – something fast, skilled, and automatic that builds upon highly specialized mechanisms dedicated to performing specific subroutines, such as retrieving appropriate words, generating morpho-syntactic structure, computing the phonological target shape of syllables, words, phrases and whole utterances, accessing their orthographic codes, and creating and executing motor programs for skilled typing. In such imitative redintegration, we might expect probabilistic effects to be at their strongest. Formulaic language is more common in speech than in writing (Erman and Warren, 2000); and the observation that memorized clauses and clause-sequences form a high proportion of the fluent stretches of speech heard in everyday conversation led Pawley and Syder (1983) to propose that it is this use of memorized language that underpins fluency.

### Method

#### Participants

Participants were Chinese native speakers (*n* = 22) who were international students at a major university in the United States. They were either sampled from the Subject Pool of the Psychology Department and participated for course credit (*n* = 1) or recruited through recruitment posters around the campus and paid $15 for their participation (*n* = 21). The sample were unintendedly female-dominant (*n* = 18). Participants were between 18 and 28 years old (Mean = 22, *SD* = 2.64). All but one of them had lived in an English immersed environment for some time^4^. Excluding this participant, the length of residence in an English-speaking country was between 6 and 84 months (Mean = 31.33, *SD* = 21.69). All participants had a high-level English proficiency sufficient to permit them to follow the English instructions and complete the language task entirely in English. Their proficiency in English was assessed by self-ratings and self-reported TOEFL scores. One participant was excluded from analysis due to excessive missing data. Summary of participant characteristics is reported in Section 1.1 of Supplementary Data Sheet 1.

#### Materials

##### Elicited imitation task

We followed the EIT design features outlined by Ortega et al. (2002). All sentences were within the recommended syllable length of 10–17 syllables to ensure optimal difficulty; the words that contain the target morpheme were placed in the middle of sentences, with filler words at the very beginning and ends of the sentences to reduce primacy and recency effects. So, in the stimulus sentence *“Late Wednesday evening I thanked him for the lovely flowers,”* the target morpheme is the *-ed* in *thanked* in the middle of the sentence, the controlled four-word context was *I thanked him for*, the primacy filler was a randomly selected three word phrase *Late Wednesday evening*, and the recency filler was *the lovely flowers*. More detail on how these sentences were constructed is described in the following section. To reduce the impact of phonological rehearsal in short term memory, a 3–5 s distraction task was set up in-between the stimulus and response for each sentence, during which the participants had to judge whether the sentence seemed sensible to them, thus reducing the opportunity for rehearsal. This semantic judgment task helped to ensure that participants actively engaged in semantic processing of the sentences rather than simply trying to encode and retain their acoustic forms. The following section describes the procedure for how the sentences were created.

##### Item development

The study targeted four of the most studied inflectional morphemes in English verbs and nouns: the regular past-tense ending *-ed*, the progressive marker *-ing*, the third-person singular present-tense ending *-s*, and nominal plural marker *-s*. Thirty sentences were made for each morpheme, which were divided into three Availability-Reliability Distribution (ARD) groups on the basis of corpus analysis in the Corpus of Contemporary American English (COCA) (Davies, 2008)^5^. COCA is widely used and frequently updated and contains over 520 million words (with 20 million words added each year from 1990 to 2015) coming from 220 thousand text sources that are equally divided among different genres of American English such as spoken, news and magazines, academic texts, fiction, etc., making it the only large and balanced corpus of English used in the United States.

**FIGURE 1:**
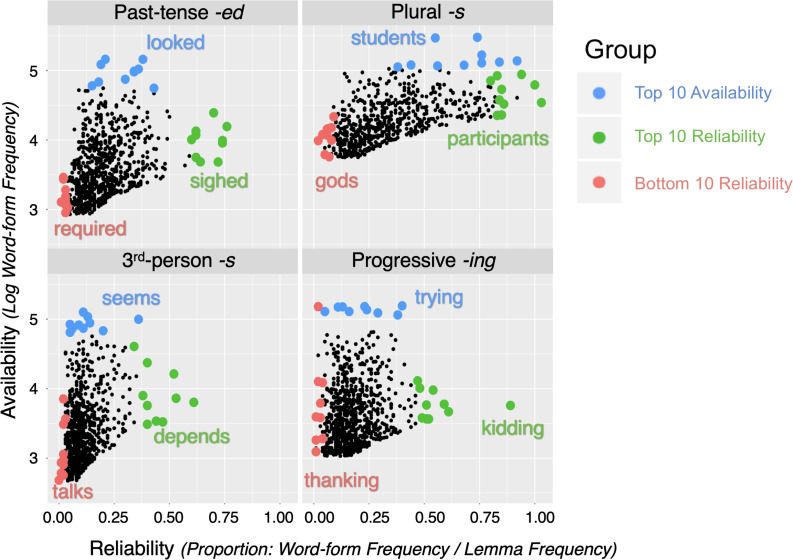
Distributional characteristics of the stimulus sample for the four morphemes: past-tense -ed **(top left)**, plural *-s* **(top right)**, third-person *-s* **(bottom left)**, and progressive *-ing* **(bottom right)**, with one example of each morpheme in each group. Dots represent the top 1000 content verbs for each morpheme. The blue dots represent stimuli belonging to the Top 10 Availability group, green dots the Top 10 Reliability group, and red dots the Bottom 10 Reliability group.

The ARD groups for each morpheme were determined on the basis of their carrier words. The three groups are: (1) Top 10 Availability, (2) Top 10 Reliability, and (3) Bottom 10 Reliability. We first assessed the lemma frequency and the inflected word-form frequency by conducting searches for the top 1000 most frequent content verbs *([vv^∗^])* and the top 1000 content nouns *([nn^∗^])* and recording the frequency counts in Excel. The Top 10 Availability group consists of the top 10 most frequent inflected word-forms exemplifying each of the four target morphemes. The search commands were as follows: regular past-tense verbs (*-ed: ^∗^ed.[vv^∗^d]*), third-person singular present-tense verbs (*-s: ^∗^s.[vv^∗^z]*), progressive verbs (-*ing*: *[vv^∗^g]*), and regular plural nouns (*-s*: *^∗^s.[^∗^nn2^∗^]*)^6^. Morpheme reliability was operationalized as the proportion of times that the lemma occurred with that specific morpheme by dividing the word-form frequency (i.e., the frequency of the word-form inflected with the target morpheme) by the lemma frequency (i.e., frequency of all possible word-forms of the lemma). For each morpheme, we ranked the items by reliability and took the top 10 of these to form the Top 10 Reliability group, unless the item had already been included in the Top 10 Availability group. Lastly, the Bottom 10 Reliability group were the items lowest in reliability of expression of the embedded morpheme. It was formed from the bottom results of the proportion rankings that were also relatively low in word-form frequency. Where there was room for choice between exemplars, we favored the alternative with the highest lemma frequency. We also tried to match the Top 10 Reliability and Bottom 10 Reliability items for word-form frequency. The frequency and reliability characteristics of the stimulus sample are summarized in Table 1. Figure 1 illustrates the distribution of the stimuli belonging to each group for each morpheme within the top 1000 frequent content verbs or nouns accordingly. We show the Top 10 Availability group in blue and illustrate with the leading exemplar (e.g., *students* for plural -s), the Top 10 Reliability group in green (*participants*), and the Bottom 10 Reliability group in red (*gods*).

**TABLE 1:** The mean lemma frequency, inflected word-form frequency (availability), and word-form:lemma proportion (reliability) of the carrier words in the stimulus sample by ARD group and by morpheme.

Group and morpheme	Lemma frequency (Mean)	Inflected word-form frequency (Mean)	Reliability^1^ (Proportion) (Mean)
Top 10 Availability	744806	121345	0.32

Past-tense *-ed*	372980	96811	0.29
Third-person *-s*	884702	86873	0.13
Progressive *-ing*	1462384	139036	0.20
Plural *-s*	259157	162659	0.66

Top 10 Reliability	28908	20042	0.64

Past-tense *-ed*	16206	10967	0.68
Third-person *-s*	29185	11750	0.45
Progressive *-ing*	12426	6597	0.56
Plural *-s*	57813	50856	0.88

Bottom 10 Reliability	815824	16912	0.03

Past-tense *-ed*	64125	1615	0.03
Third-person *-s*	97507	1929	0.02
Progressive *-ing*	2904224	53365	0.02
Plural *-s*	197441	10741	0.06

*^1^Reliability = Word-form frequency/Lemma frequency.*

To build the sentence contexts for these carrier words, we first conducted n-gram searches for possible three-word strings with the target word in the middle (e.g., *[^∗^ wanted ^∗^]*) and then selected the top frequent results for each. These results (e.g., *[you wanted to]*) were then fed into searches for possible four-word strings with an extra slot at the end (i.e., *[you wanted to ^∗^]*). Then, a three-word random time filler phrase (e.g., *On Wednesday morning…*) was put at the beginning of each sentence. We only included tense-neutral time phrases — those that do not provide any lexical time cue — so that the target inflectional morpheme would be the only indicator of the tense, i.e., the morphemes would be in non-obligatory contexts. The sentences were then completed to a length of 14–17 syllables by adding a random possible phrase at the end that would make the whole sentence grammatical, logical, and relatively sensible. All filler words were checked with a lexical range breakdown using the computer program VocabProfile from the Compleat Lexical Tutor website (Cobb, accessed 8/2017) so they are roughly in the same frequency band, mostly the top 1000 frequent words. All the finalized sentences were manually checked by two native Chinese speakers to make sure they were sufficiently comprehensible for an average Chinese ESL learner. All sentences were recorded in Audacity^7^. Each of them was spoken twice by a male native speaker of American English and was evaluated by another native speaker to select the best version. Sample sentences can be found in Table 2. The complete stimuli set including the full list of words with their lemma frequency, word-form frequency, and the calculated reliability along with their carrier sentences is available in Supplementary Data Sheet 2.

**TABLE 2:** Sample sentences for selected morpheme in each group for Experiment 1.

Morpheme frequency group	Target morpheme	*Sentence*	Word-form frequency	Lemma frequency	Proportion (reliability)
Top 10 availability	*-ed*	*On Monday afternoon he* ***looked*** *at me for a long moment*	121996	652141	0.19
Top 10 reliability	*-ing*	*Late Thursday evening I was* ***kidding*** *about his hair and beard*	5734	6410	0.89
Bottom 10 reliability	3sg *-s*	*On Friday afternoon he* ***talks*** *about the latest developments*	7125	304560	0.02
Bottom 10 reliability	Plural *-s*	*On Wednesday she hears about the* ***gods*** *of the new religion*	6156	125937	0.05

*Target word in each sentence is bolded.*

### Procedure

The EIT task was administered individually through a PsychoPy program (PsychoPy, RRID:SCR_006571, Peirce, 2007) running on an iMac computer equipped with headphones located in an experimental booth. The total duration of the experiment was approximately 80 min. After providing informed consent, participants received brief oral instructions from the experimenter. The program began with an instruction screen that explained how each sentence would be presented and what their task was, followed by a practice session of five sentences. Participants proceeded to the experimental trials if no further questions arose. The experimenter remained available to aid them as needed.

All participants listened to the 120 sentences. The presentation sequence was individually randomized. On each trial, they first heard a spoken sentence, such as *“Late Wednesday evening I thanked him for the lovely flowers.”* Immediately after the audio ended, the screen displayed instructions for the participant to judge how much sense the sentence made to them by rating it on a sliding scale of 1–7 using the mouse. Once this rating had been completed, the participant was asked to type out the complete and correct sentence to the best of their ability. Participants decided when the next trial should start by pressing the spacebar. They were notified at the midpoint of the experiment and allowed to take a short break if desired. Their reproduction of the sentences was recorded in csv files.

After the experiment, participants completed a 5-min language history questionnaire (Supplementary Data Sheet 1, 2.2) adapted from Lim and Godfroid (2015). This included questions on general demographics such as gender and age, as well as language background including previous and current exposure and usage of English, English proficiency test scores, self-rated general proficiency in English, and self-rated proficiency on different aspects of using English (reading, writing, speaking, and listening).

Elicited imitation responses were scored for accuracy of production of the one target word that contained the target morpheme in each sentence in the following steps: First, using a string search command in Excel, we screened whether each response contained the exact match of the target word (marked as 1) or not (marked as 0). “Exact matches” were also automatically marked 1 for “correct lemma” and 1 for “correct morpheme.” Second, we manually checked responses marked as 0s for “exact match” looking for typos and spelling errors, as well as irregularities in the inflectional paradigms of certain words, to decide whether a reasonable *attempt* at the target word was present. In cases where the attempted target word reasonably resembled any form of the lemma (e.g., *^∗^glansed* for *glanced*), they were given a “correct lemma” score of 1. Likewise, if its ending reasonably resembled the target morpheme (e.g., *^∗^lookign* for looking), or if its form resembled the tense or number indicated by the target morpheme (e.g., *^∗^drooling* for drilling), they were given a “correct morpheme” score of 1. Finally, using Excel commands again, we identified whether the correct morpheme was present in the target word *given* the presence of a correct lemma (“correct morpheme *given* correct lemma”). To sum up, each typed response was either marked as 1 (for “correct morpheme *given* correct lemma”), 0 (for “incorrect or absent morpheme *given* correct lemma”), or N/A (for cases where the lemma is absent). The lemma-absent cases constituted 8.91% of the responses and were excluded from further analysis. The scoring method is illustrated in Table 3 with two examples of each scenario.

**TABLE 3:** Scoring method with sample sentences.

	Correct morpheme *given* correct lemma	Score: 1
*Examples:*	*(1a) Late Wednesday evening, I* ***thanked*** *him for the lovely flowers.*	
	*(2a) On Thursday, she knew about the* ***fathers*** *of the fat children.*	

	**Incorrect or absent morpheme *given* correct lemma**	**Score: 0**

*Examples:*	*(1b) Late Wednesday evening, I* ***thanks*** *him for the lovely flowers.*	
	*(2b) On Thursday, she knew about the* ***father*** *of the fat children.*	

	**Incorrect or absent lemma**	**Score: N/A (excluded)**

*Examples:*	*(1c) Late Wednesday evening, I* ***think*** *him for the lovely flowers.*	
	*(2c) On Thursday, she knew about the* ***mothers*** *of the fat children.*	

*Target word in each sentence is bolded.*

### Results

**FIGURE 2:**
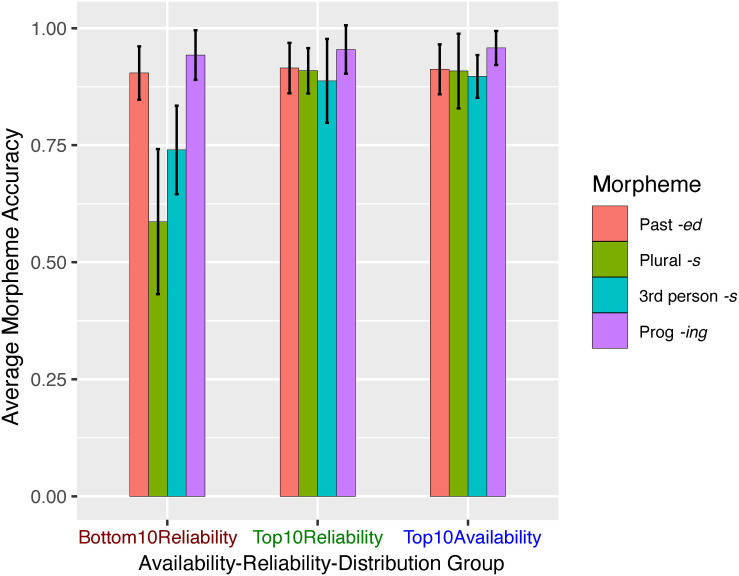
Experiment 1 mean accuracy scores (correct morpheme | correct lemma) by ARD group and morpheme. Error bars represent ± 1 SE.

The accuracy scores of sentences for each morpheme in each ARD group are shown in Figure 2. To examine the effects of the two distributional factors, availability and reliability, on the production accuracy of the morphemes, we used generalized linear mixed-effect models using the “lme4” package (R package: lme4, RRID:SCR_015654, version 1.1-13, Bates et al., 2015) in R (version 3.3.3, R Core Team, 2017). The models were fit by maximum likelihood (Laplace Approximation), with random effects specified for subjects and items. Because the four target morphemes are inherently stratified in frequency in the corpus, e.g., past-tense *-ed* verbs are generally used more frequently than third-person *-s* verbs, the distributional factors are correlated with morpheme type. To reduce multicollinearity and to account for the between-morpheme differences, we first ran a mixed-effect model with morpheme type as the only fixed-effect predictor to serve as a baseline model which parses out the differences between the four morphemes. From there, we built up the model by incrementally specifying other predictors one at a time to identify the unique contributions of each. To determine which subject-level random effects to include, specifically whether or not to include the random slopes for subjects for each fixed-effect predictor, we ran two versions for each model, one with only random intercepts, and one further adding random slopes. We report the model with random intercepts for subjects unless adding random slopes significantly improves model fit, in which case we report the latter. The preliminary steps (testing morpheme type alone; morpheme type + morpheme reliability; and morpheme type + morpheme availability) are detailed in Supplementary Data Sheet 1, 1.2. We describe here the complete Model 1 involving all three fixed effects.

#### Model 1: Morpheme Type + Morpheme Reliability + Morpheme Availability

Model 1, which included morpheme type, reliability, availability (i.e., log word-form frequency) as fixed-effect predictors, and random intercepts for subjects and items), is detailed in Table 4. Stimulus sentence length in syllables was included as a fixed predictor to control for any stimulus length effects. Each participant’s stimulus sense rating for each sentence was also included as a potential predictor.

**TABLE 4:** Experiment 1 results from the mixed effects model including fixed effects of morpheme type, morpheme reliability (proportion), morpheme availability (log word-form frequency), stimulus sentence length, stimulus sense rating, and random effects of subject and item.

Model 1: no interactions.							

		*Fixed effects*				*Random effects*	
		
						By subject	By item
	
*Parameters*		Estimate	*SE*	*z*	*p*	*SD*	*SD*
Intercept		0.145	3.424	0.04	0.966	1.04	1.24
Morpheme type^1^	Plural -s	–1.480	0.424	–3.49	0.000***		
	Third-person -s	–0.347	0.391	–0.88	0.376		
	Progressive -ing	0.963	0.435	2.21	0.027*		
Morpheme reliability		1.937	0.536	3.61	0.000***		
Morpheme availability^2^		0.420	0.202	2.08	0.037*		
Sentence length (syllables)		0.035	0.207	0.17	0.866		
Sense rating		–0.009	0.059	–0.16	0.874		

^1^Past-tense *-ed* is the reference level. ^2^Word-form frequency was logarithmically transformed. Model formula: accuracy∼morpheme + reliability + availability + length + sense + (1| subject) + (1| item). ***p = 0; *p < 0.01; *p* < 0.1.							

**Model 1b: Interactions between morpheme type and reliability.**							

		***Fixed effects***				***Random effects***	
		
						**By subject**	**By item**
	
***Parameters***		**Estimate**	***SE***	***z***	***p***	***SD***	***SD***

Intercept		3.491	2.940	1.19	0.235	1.10	1.11
Morpheme type^1^	Plural -s	–2.667	0.593	–4.50	0.000***		
	Third-person -s	–1.257	0.536	–2.35	0.019*		
	Progressive -ing	0.534	0.594	0.90	0.368		
Morpheme reliability		0.554	1.026	–0.54	0.589		
Sentence length (syllables)		–0.028	0.192	–0.14	0.886		
MorphemePlural-s: Reliability		3.902	1.231	3.17	0.002**		
MorphemePres-s: Reliability		2.952	1.638	1.80	0.072.		
MorphemeProg-ing: Reliability		1.626	1.647	0.99	0.323		

^1^Past-tense *-ed* is the reference level. Model formula: accuracy∼morpheme^∗^reliability + length + (1| subject) + (1| item). ***p = 0; **p < 0.001; *p < 0.01; ^⋅^*p* < 0.05; *p* < 0.1.							

**Model 1c: interactions between morpheme type and availability.**							

		***Fixed effects***				***Random effects***	
		
						**By Subject**	**By Item**
	
***Parameters***		**Estimate**	***SE***	***z***	***p***	***SD***	***SD***

Intercept		1.765	3.546	0.50	0.619	1.10	1.15
Morpheme type^1^	Plural -s	–9.827	2.673	–3.68	0.000***		
	Third-person -s	–2.660	1.869	–1.42	0.155		
	Progressive -ing	1.779	2.246	0.79	0.428		
Morpheme availability^2^		0.221	0.355	0.62	0.534		
Sentence length (syllables)		0.017	0.200	0.08	0.934		
MorphemePlural-s: Availability		1.943	0.604	3.22	0.001**		
MorphemePres-s: Availability		0.517	0.458	1.13	0.259		
MorphemeProg-ing: Availability		–0.201	0.527	–0.38	0.703		

^1^Past-tense -ed is the reference level. ^2^Word-form frequency was logarithmically transformed. Model formula: accuracy∼morpheme^∗^availability + length + (1| subject) + (1| item). ***p = 0; **p < 0.001; p < 0.1.

There were effects of morpheme type: *-ing* had significantly higher accuracy than *-ed* (estimate = 0.963, *SE* = 0.435, *z* = 2.21, *p* = 0.027); Plural *-s* had significantly lower accuracy than *-ed* (estimate = –1.480, *SE* = 0.424, *z* = –3.49 *p* = 0.000). The difference between the third person present tense *-s* and *-ed* was not significant (estimate = –0.347, *SE* = 0.392, *z* = –0.88, *p* = 0.37). The effect of morpheme reliability was highly significant (estimate = 1.937, *SE* = 0.537, *z* = 3.61, *p* = 0.000). Additionally, availability had significant but smaller effects (estimate = 0.419, *SE* = 0.201, *z* = 2.08, *p* = 0.037). Stimulus sentence length was non-significant (estimate = 0.035, *SE* = 0.207, *z* = 0.17, *p* = 0.866). Stimulus sense rating was also non-significant (estimate = –0.009, *SE* = 0.059, *z* = –0.16, *p* = 0.874).

**FIGURE 3:**
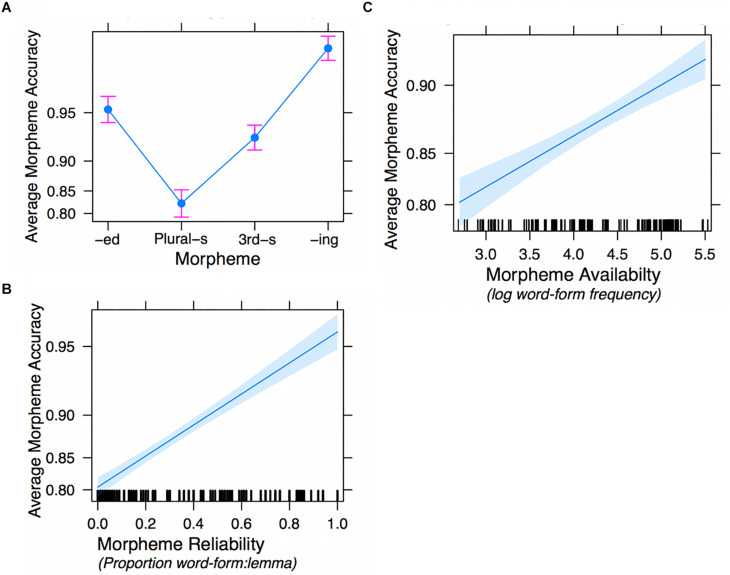
Effect plots of Model 1d fixed-effect predictors: morpheme type **(A)**, morpheme reliability **(B)**, and morpheme availability **(C)**.

Analysis of Deviance using Type III Wald chi*-*square tests showed that morpheme type, reliability, and availability were all significant predictors of accuracy [morpheme type: χ^2^(df = 3) = 31.595, *p* = 0.000; reliability: χ^2^(df = 1) = 13.015, *p* = 0.000; availability: χ^2^(df = 1) = 4.332, *p* = 0.04], confirming their individual unique contributions to production accuracy. Stimulus sentence length was not a significant predictor χ^2^(df = 1) = 0.055, *p* = 0.814, nor was stimulus sense χ^2^(df = 1) = 0.025, *p* = 0.874. Figure 3 separately plots the effects of Morpheme (3a), reliability (3b), and availability (3c).

Model 1b (mid panel of Table 4) investigated the interaction between morpheme type and reliability. Past-tense *-ed* was the reference level for type. Allowing for the interaction removes any overall effect of reliability (estimate = 0.554, *SE* = 1.026, *z* = –0.54, *p* = 0.589). However, there remains a significant effect of reliability on Plural *-s* (estimate = 3.902, *SE* = 1.231, *z* = 3.17, *p* = 0.002) and a marginal one on third person present tense *-s* (estimate = 2.952, *SE* = 1.638, *z* = 1.80, *p* = 0.072).

Model 1c (lower panel of Table 4) investigated the interaction between morpheme type and availability, again with past-tense *-ed* as the reference level. Allowing for the interaction removes any overall effect of availability (estimate = 0.221, *SE* = 0.355, *z* = 0.62, *p* = 0.534); although there remains a substantial effect of availability upon Plural *-s* (estimate = 1.943, *SE* = 0.604, *z* = 3.22, *p* = 0.001).

#### Exploring Log Lemma Frequency

To examine any effects of lemma frequency (rather than the frequency of the inflected form) alongside morpheme type, we ran a model which included morpheme type and log lemma frequency as fixed-effect predictors and random intercepts for subjects and items. Morpheme type showed consistent effects: *-ing* had significantly higher accuracy than *-ed* (estimate = 1.031, *SE* = 0.481, *z* = 2.14, *p* = 0.03); Plural *-s* had significantly lower accuracy than *-ed* (estimate = –0.875, *SE* = 0.431, *z* = –2.03, *p* = 0.04). However, the effect of log lemma frequency was negligible (estimate = –0.007, *SE* = 0.223, *z* = –0.03, *p* = 0.98). Analysis of Deviance using Type III Wald chi*-*square tests confirmed the morpheme effect [χ^2^(df = 3) = 21.231, *p* = 0.000], and revealed that lemma frequency was not a significant predictor of accuracy when morpheme type was taken into account [χ^2^(df = 1) = 0.001, *p* = 0.97].

#### *Post hoc* Explorations of Effects of n-Gram Frequency

As a *post hoc* analysis to explore potential effects of phrasal frequency, we investigated whether log frequency of the three-word string (e.g., *you wanted to*, see section “Item Development”) explained significant additional variance alongside morpheme type. Morpheme type showed consistent effects: *-ing* had significantly higher accuracy than *-ed* (estimate = 1.131, *SE* = 0.445, *z* = 2.54, *p* = 0.01); Plural *-s* had significantly lower accuracy than *-ed* (estimate = –0.980, *SE* = 0.401, *z* = –2.44, *p* = 0.02). The effect of log 3-gram frequency was also highly significant (estimate = 0.526, *SE* = 0.165, *z* = 3.18, *p* = 0.001). Analysis of Deviance using Type III Wald chi*-*square tests showed that in addition to morpheme type, log 3-gram frequency was also significantly predictive of accuracy [morpheme type: χ^2^(df = 3) = 26.66, *p* = 0.000; log 3-gram frequency: χ^2^(df = 1) = 10.14, *p* = 0.001].

To try to see whether availability (i.e., log word-form frequency) or log 3-gram frequency had independent effects, and which was the greater contributor, we tried models which included both as potential contributors. However, because log word-form frequency and log 3-gram frequency were inherently highly correlated (*r* = 0.810), they pull against each other and neither ends up as significant: availability (estimate = 0.025, *SE* = 0.345, *z* = 0.07, *p* = 0.941), log 3-gram frequency (estimate = 0.425, *SE* = 0.274, *z* = 1.55, *p* = 0.122). This is to be further investigated in Experiment 2.

#### Results Summary

In sum, these analyses revealed independent effects on production accuracy of morpheme availability and reliability. The interactions of these factors with morpheme type revealed a significant effect of reliability on Plural *-s* and a marginal effect of third person present tense -*s*, and significant effects of availability on Plural -*s.* In contrast to availability of the inflected form, there were no effects of log lemma frequency. Neither sentence length nor sense rating had any effect on morpheme provision. Post-hoc exploratory analyses showed that the frequency of the three-word strings also positively predicted accurate provision of the embedded morpheme. However, we had not planned this analysis and had not systematically manipulated the 3-gram frequency in the stimulus materials or controlled for the inherently high correlation between the frequency of the three-word string and the frequency of the word-form inside the string. More careful controls of string frequency are therefore needed to confirm this tentative conclusion.

### Discussion of Experiment 1

As predicted, both availability and reliability of the morphemes were positively associated with morpheme production accuracy in the EIT. A morpheme (e.g., plural-*s*) in a word-form (e.g., *participant-s*) is more easily recognized and produced when the word-form is high in token frequency and when it is the more reliable form of the lemma ([PARTICIPANT]). The effects of reliability were numerically greater than those of availability.

The participants showed a greater sensitivity to the distribution of the morphologically complex surface forms of the words than to the distribution of the underlying lemmas. This finding supports those of Bybee (1985) and Hay (2001) on the importance of relative frequency in derivational morphology described in the introduction. Similar patterns were also observed in Sereno and Jongman’s (1997) lexical decision task, in which words were presented in singular (e.g., *car*), or in plural (*cars*) to native speakers. It was found that the difference in reaction times were predicted only by how frequent the specific surface form was presented, whether singular or plural, but not by the total frequency of both forms (i.e., the lemma frequency). Sereno and Jongman took this as evidence against rule-based processing models of inflectional morphology.

The rank order difficulty of the target morphemes (*-ing* > *-ed* > third person present tense -*s* > plural-*s*) was generally consistent with the common order reported in prior SLA morpheme studies (Krashen et al., 1977; Goldschneider and DeKeyser, 2001):


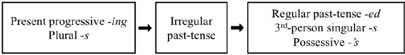


with the exception of the plural *-s*, which was previously reported to be among the earliest to be acquired and processed by L1 and L2 learners of English (Brown, 1973; Krashen et al., 1977). Due to the limited sample size, we refrain from further interpreting this pattern unless it is replicated in Experiment 2. Note also that our stimuli involve a systematically factored selection of 30 exemplars of each type rather than a representationally random sample as used in previous studies, and this might have led to the deviation from the common order.

*Post hoc* exploratory analyses involving the three-word string suggested that frequency beyond the lexical level could also have affected the production accuracy of the embedded morpheme. As previously discussed, facilitation effects of string frequency (formulaicity) have been observed in the processing of phrasal expressions and non-phrasal “lexical bundles” (Arnon and Snider, 2010; Tremblay et al., 2011), and high frequency frames can facilitate the acquisition and processing of individual component words (Childers and Tomasello, 2001). However, formulaicity research has primarily focused on the facilitation effects on the processing and acquisition of lexical items (Ellis, 2012b; Siyanova-Chanturia and Pellicer-Sanchez, 2018) rather than morphology. The demonstration of effects of formulaicity upon L2 morpheme processing requires more formal control and investigation in a design with greater power than the post-hoc explorations we report here – hence Experiment 2.

## Experiment 2

Here we aimed to replicate Experiment 1’s findings on the pattern of morpheme acquisition, and the facilitation effects of morpheme availability and reliability, with a larger sample of participants, with improved stimulus materials, and with a new speaker for the stimulus recordings. Importantly, it extended to the investigation of the effects on morpheme processing and production of frequency at a phrasal level, i.e., the frequency of the four-word strings that contained the target morpheme. To achieve this, we included the same morphemes as those in Experiment 1 but embedded them in high- and low- frequency four-word strings in the sentences for elicited imitation. Motivated by existing literature on formulaicity and the preliminary results from Experiment 1, we predicted that besides the frequency of the word-forms inflected with the target morpheme, the frequency of the four-word strings in which the morpheme-carrying word-form are embedded would also positively predict the morpheme production accuracy in the elicited imitation of sentences.

### Method

#### Participants

Forty-nine native Mandarin Chinese speakers who did not participate in Experiment 1 were recruited for Experiment 2. They were sampled from the same population as the participants in Experiment 1 and were recruited with the same poster. They were paid $15 for their participation. Data from four participants were excluded from the analysis due to computer malfunction (*N* = 3) or incompletion of the task (*N* = 1). The remaining 45 participants were predominantly female (*N* = 29), and were between 18 and 28 years old (Mean = 22.02, *SD* = 3.20). All of them have been exposed to an English immersion environment. The length of residence in an English-speaking country was between 0.5 and 192 months (Mean = 44.28, *SD* = 48.15). All participants were sufficiently proficient to complete the task in English. Their English proficiency was assessed by self-ratings and self-reported TOEFL scores using the questionnaire of Experiment 1. Participant characteristics for Experiment 2 are reported in Section 1.3 of Supplementary Data Sheet 1.

#### Materials

The materials used in the EIT were similar to those used in Experiment 1. All the morphemes and target words remained the same. Experiment 2 had a 4 (morphemes) ^∗^ 3 (morpheme ARD groups) ^∗^ 2 (string frequency groups) design. The string frequency grouping factor was operationalized using the COCA-based frequency of the four-word string in which the target morpheme embedded. For example, for sentence 1a in Table 3, where *‘‘thanked’’* is the target word for the target morpheme *-ed*, the four-word string is *‘‘I thanked him for,’’* which occurred 56 times in COCA. Thus, the string frequency for this item is 56. The 120 stimulus sentences in Experiment 1 had been selected for high 4-gram frequency (i.e., the frequency of the four-word string) by design. Thus, we included the Experiment 1 sentences as the *a priori* high string frequency group^8^.

To form the low string frequency group, 120 additional sentences were created by modifying the four-word strings that contain the target morphemes in the 120 existing ones. For the three verbal morphemes (*-ed*, *-ing*, and third-person *-s*), we adopted a modified version of the manipulation of Childers and Tomasello (2001) and constructed the low string frequency version by substituting any pronouns in the high frequency string with person names, e.g., changing *‘‘I thanked him for’’* into *‘‘Ashley thanked Steven for.’’* The resulting low string frequency sentences contained either one or two person names depending on the transitivity of the target verb. All person names were highly familiar^9^ and were between 1 and 4 syllables long. Nevertheless, the resulting low string frequency 4-grams typically do not occur in COCA, resulting in a 4-gram frequency of 0.

For the sentences containing the noun plural *-s*, in which the high frequency four-word strings did not typically contain pronouns, the low string frequency versions were constructed by inserting a familiar adjective before the target word that contained the morpheme. In the example sentence 2a given in Table 3, in which *“the fathers of the”* was the high frequency four-word string containing the plural *-s*, we inserted the adjective *“real”* before *“fathers”* so that the low string frequency four-word string became *“real fathers of the.”* We made sure that the adjective-noun pairs were high in bigram frequency to avoid any syntactic violations and/or semantic oddity. We also made sure that all the inserted adjectives were highly frequent words themselves and were all 1–2 syllables long so that the two versions of the sentences were comparable in length. On average, the low string frequency sentences were 1.175 syllables longer than their high frequency counterparts.

All 240 sentences were between 14 and 17 syllables. They were recorded by a female native speaker of American English in a noise-proof recording booth. In order to avoid familiarity effects, we made two counterbalanced versions of the stimuli: the first had odd items (1, 3, 5…) of high frequency and even items (2, 4, 6…) of low frequency, and vice versa for the second version. In this way, subjects never encountered both versions of a matched pair (For full stimuli list, see Supplementary Data Sheet 2).

#### Procedure

The procedure remained the same as in Experiment 1. The total duration of Experiment 2 was approximately 80 min. For each participant, the 120 sentences were presented in an individually randomized sequence. The two versions were interspersed in participants to ensure equal number of participants completing each version. The typed responses were recorded in .csv files for analysis. The scoring method also remained the same: correct morpheme given the presence of a correct lemma was marked as 1; incorrect or absent morpheme given correct lemma was marked as 0; lemma-absent cases were marked as N/A and were excluded from further analysis (17.19%).

### Results

#### Model 1: Morpheme Type + Morpheme Reliability + Morpheme Availability

**FIGURE 4:**
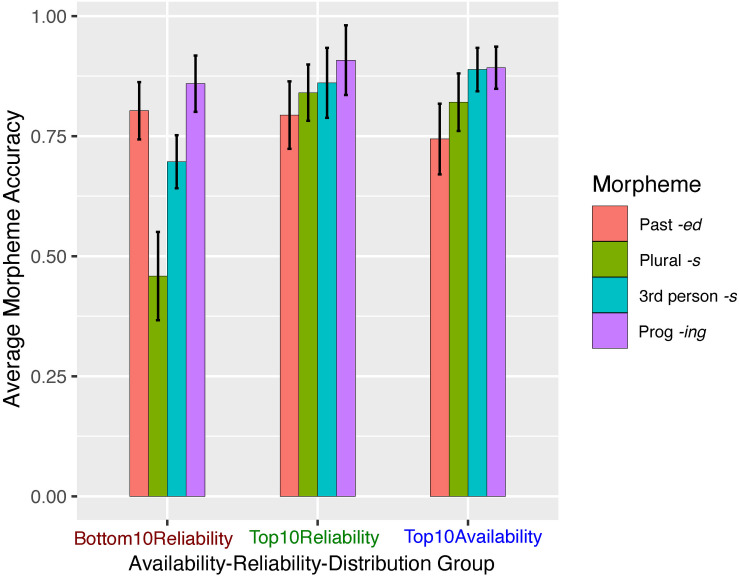
Experiment 2 mean accuracy scores (correct morpheme | correct lemma) by ARD group and morpheme type. Error bars represent ± 1 SE.

The accuracy scores for sentences by morpheme and ARD group are shown in Figure 4. To replicate Experiment 1, we first conducted GLMM analyses without formulaicity as a factor. The results closely mirrored those of Experiment 1 Model 1, demonstrating significant effects of morpheme type, reliability and availability (see Supplementary Data Sheet 1, 1.4). In this sample there was reason to include subject random slopes for both reliability [χ^2^(df = 2) = 7.754, *p* = 0.02] and availability [χ^2^(df = 3) = 314.7, *p* = 0.000]^10^.

#### Model 2: Morpheme Type + Morpheme Reliability + Morpheme Availability + Formulaicity

**FIGURE 5:**
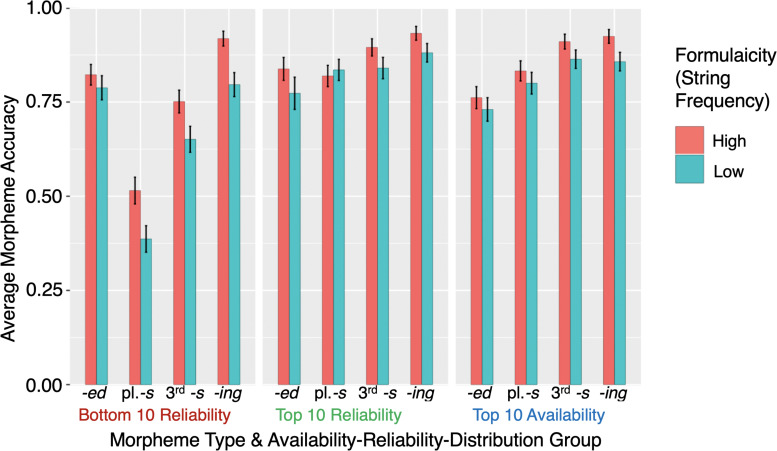
Experiment 2 mean accuracy scores (correct morpheme | correct lemma) by ARD group, morpheme type, and string frequency group. Error bars represent ± 1 SE.

The accuracy scores for sentences by morpheme, ARD group, and formulaicity are shown in Figure 5. To examine any additional effects of formulaicity, we added string frequency as another fixed-effect predictor and conducted incremental model comparisons to determine a maximal model which included fixed-effects of morpheme type, morpheme reliability, morpheme availability, and formulaicity, random intercepts for subjects and items, and random slopes by subject for morpheme reliability and morpheme availability. In creating the sentence stimuli for Experiment 2, formulaicity was inevitably correlated with sentence length (on average, the low string frequency sentences were 1.175 syllables longer than their high frequency counterparts). To rule out the possibility that sentence length is what drives any observed formulaicity effect, as we did in Experiment 1 (where sentence length was non-significant), we included sentence length (i.e., total number of syllables) as a fixed-effect predictor of morpheme production accuracy. Each participant’s stimulus sense rating for each sentence was also included as a potential predictor; model comparison using a likelihood ratio test showed no need to include subject random slopes for sense rating [*χ*^2^(df = 6) = 0.911, *p* = 0.99). The final model is summarized in Table 5. To see the random slopes for effects of availability and reliability in each subject are shown, refer to Supplementary Data Sheet 1, 3.1).

**TABLE 5:** Experiment 2 including fixed-effects of morpheme type, morpheme reliability (proportion), morpheme availability (log word-form Frequency), formulaicity (string frequency), length (syllables), and stimulus sense rating, and random effects of subject and item.

Model 2: no interactions.							

		*Fixed effects*				*Random effects*	
		
						By subject	By item
	
*Parameters*		Estimate	*SE*	*z*	*p*	*SD*	*SD*
Intercept		0.484	2.135	–0.23	0.820	3.027	1.048
Morpheme type^1^	Plural *-s*	–0.875	0.239	–3.66	0.000***		
	Third-person *-s*	0.809	0.236	3.42	0.001***		
	Progressive *-ing*	1.293	0.246	5.25	0.000***		
Morpheme reliability		1.742	0.348	5.01	0.000***	0.786	
Morpheme availability^2^		0.301	0.126	2.39	0.017*	0.026	
Formulaicity (low)^3^		–0.619	0.223	–2.77	0.006**		
Sentence length (syllables)		–0.014	0.129	–0.11	0.921		
Sense rating		–0.126	0.035	–3.65	0.000***		

^1^The Past-tense -ed is the reference level. ^2^Word-form frequency was logarithmically transformed. ^3^High formulaicity was the reference level. Model formula: accuracy∼morpheme + reliability + availability + formulaicity + length + sense + (1 + reliability + availability| subject) + (1| Item). ***p = 0; **p < 0.001; *p < 0.01; *p* < 0.1.							

**Model 2b: Interactions between morpheme type and reliability.**							

		***Fixed effects***				***Random effects***	
		
						**By subject**	**By item**
	
***Parameters***		**Estimate**	***SE***	***z***	***p***	***SD***	***SD***

Intercept		4.294	1.886	2.28	0.023*	1.288	0.955
Morpheme type^1^	Plural -s	–2.249	0.352	–6.38	0.000***		
	Third-person -s	–0.059	0.32	–0.18	0.853		
	Progressive -ing	0.743	0.34	2.19	0.029*		
Morpheme reliability		–0.604	0.598	–1.01	0.312	0.960	
Formulaicity (low)^2^		–0.462	0.211	–2.19	0.028*		
Sentence length (syllables)		–0.153	0.122	–1.25	0.210		
MorphemePlural-s: Reliability		3.995	0.722	5.54	0.000***		
MorphemePres-s: Reliability		2.781	1.01	2.75	0.006**		
MorphemeProg-ing: Reliability		1.881	0.946	1.99	0.047*		

^1^Past-tense *-ed* is the reference level. ^2^ High formulaicity was the reference level. Model formula: accuracy∼morpheme^∗^reliability + formulaicity + length + (1 + reliability| subject) + (1| item). ***p = 0; **p < 0.001; *p < 0.01; *p* < 0.1.							

**Model 2c: interactions between morpheme type and availability.**							

		***Fixed effects***				***Random effects***	
		
						**By Subject**	**By Item**
	
***Parameters***		**Estimate**	***SE***	***z***	***p***	***SD***	***SD***

Intercept		2.453	2.209	1.110	0.267	1.943	0.984
Morpheme type^1^	Plural -s	–9.346	1.655	–5.650	0.000***		
	Third-person -s	–3.651	1.147	–3.180	0.001**		
	Progressive -ing	0.526	1.292	0.410	0.684		
Morpheme availability		–0.158	0.209	–0.760	0.448	0.269	
Formulaicity (low)^2^		–0.638	0.217	–2.940	0.003**		
Sentence Length (syllables)		–0.002	0.127	–0.020	0.985		
MorphemePlural-s: Availability		1.971	0.369	5.340	0.000***		
MorphemePres-s: Availability		1.051	0.281	3.740	0.000***		
MorphemeProg-ing: Availability		0.169	0.302	0.560	0.575		

^1^Past-tense -ed is the reference level. ^2^High formulaicity was the reference level. Model formula: accuracy∼morpheme*availability + formulaicity + length + (1 + availability| subject) + (1| item). ***p = 0; **p < 0.001; p < 0.1.

There were effects of morpheme type: *-ing* had significantly higher accuracy than *-ed* (estimate = 1.293, *SE* = 0.246, *z* = 5.25, *p* = 0.000); Plural *-s* had significantly lower accuracy than *-ed* (estimate = –0.875, *SE* = 0.239, *z* = –3.66, *p* = 0.000). The difference between the third person present tense *-s* and *-ed* was also significant (estimate = 0.809, *SE* = 0.236, *z* = 3.42, *p* = 0.001). The effect of morpheme reliability was highly significant (estimate = 1.742, *SE* = 0.348, *z* = 5.01, *p* = 0.000). Availability had significant but smaller effects (estimate = 0.301, *SE* = 0.126, *z* = 2.39, *p* = 0.017). Formulaicity was significant (estimate = –0.619, *SE* = 0.223, *z* = –2.77, *p* = 0.006). Stimulus sentence length was non-significant (estimate = –0.014, *SE* = 0.129, *z* = –0.11, *p* = 0.92). Stimulus sense rating was significant (estimate = 0.126, *SE* = 0.035, *z* = 3.65, *p* = 0.000).

Analysis of Deviance using Type III Wald chi-square tests shows that morpheme type, morpheme reliability (proportion), morpheme availability (log word-form frequency), formulaicity (string frequency), and sentence sense rating were all significant predictors of accuracy [morpheme type: χ^2^(df = 3) = 81.940, *p* = 0.000; morpheme reliability: χ^2^(df = 1) = 25.111, *p* = 0.000; morpheme availability: χ^2^(df = 1) = 5.724, *p* = 0.017; formulaicity: χ^2^(df = 1) = 7.688, *p* = 0.006; sentence sense rating: χ^2^(df = 1) = 13.321, *p* = 0.000]. Stimulus length was non-significant: χ^2^(df = 1) = 0.012, *p* = 0.912.

Model 2b (mid panel of Table 5) investigated the interaction between morpheme type and reliability. Past-tense *-ed* was the reference level for type. Allowing for the interaction removes any overall effect of reliability (estimate = –0.604, *SE* = 0.598, *z* = –1.01, *p* = 0.312). However, there remain significant effect of reliability on Plural *-s* (estimate = 3.995, *SE* = 0.722, *z* = 5.54, *p* = 0.000), third person present tense *-s* (estimate = 2.781, *SE* = 1.010, *z* = 2.75, *p* = 0.006), and Prog*-ing* (estimate = 1.881, SE = 0.946, *z* = 1.99, *p* = 0.047).

Model 2c (lower panel of Table 5) investigated the interaction between morpheme type and availability, again with past-tense *-ed* as the reference level. Allowing for the interaction removes any overall effect of availability (estimate = –0.158, *SE* = 0.209, *z* = –0.76, *p* = 0.448); although there remains a substantial effect of availability upon Plural *-s* (estimate = 1.971, *SE* = 0.369, *z* = 5.34, *p* = .000) and third person present tense *-s* (estimate = 1.051, *SE* = 0.281, *z* = 3.74, *p* = 0.000).

#### Model 3: Exploring Effects of Proficiency

**FIGURE 6:**
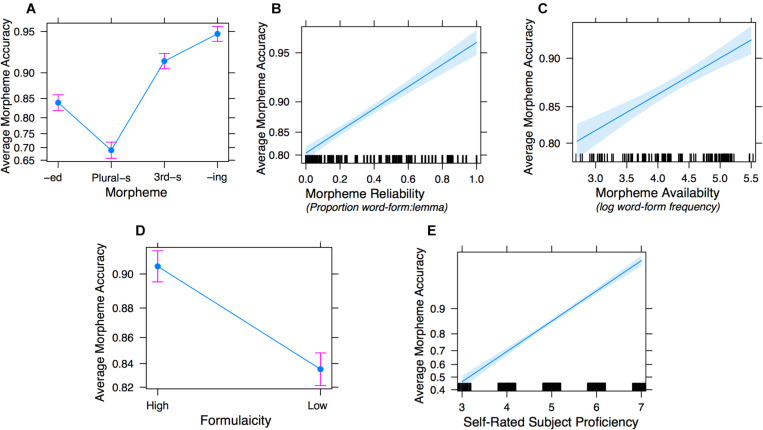
Effect plots of Model 3 fixed-effect predictors: morpheme type **(A)**, morpheme reliability **(B)**, morpheme availability **(C)**, formulaicity **(D)**, and subject proficiency **(E)**.

To test whether individual differences in English proficiency were reflected in participants’ morpheme production accuracy, we first included self-rated general proficiency scores as a fixed-effect predictor (in addition to morpheme type, reliability, availability, and formulaicity) without any interaction terms. As shown in Figure 6, the effects of morpheme type were consistent with previous models: *-ing* had significantly higher accuracy than -*ed* (estimate = 1.260, *SE* = 0.245, *z* = 5.15, *p* = 0.000); the Plural -*s* had significantly lower accuracy than -*ed* (estimate = –0.857, *SE* = 0.238, *z* = –3.60, *p* = 0.000); the third-person -*s* had significantly higher accuracy than -*ed* (estimate = 0.762, *SE* = 0.234, *z* = 3.26, *p* = 0.001). The significant effects of morpheme reliability (estimate = 1.742, *SE* = 0.341, *z* = 5.10, *p* = 0.000), availability (estimate = 0.326, *SE* = 0.122, *z* = 2.67, *p* = 0.008), and formulaicity (estimate = –0.628, *SE* = 0.165, *z* = –3.81, *p* = 0.000) all remained the same as in Model 2. Importantly, subject proficiency also positively predicted morpheme accuracy (estimate = 0.974, *SE* = 0.162, *z* = 6.02, *p* = 0.000), and the addition of subject proficiency as a fixed-effect predictor significantly improved model fit from Model 2 [χ^2^(df = 1) = 27.24, *p* = 0.000].

**FIGURE 7:**
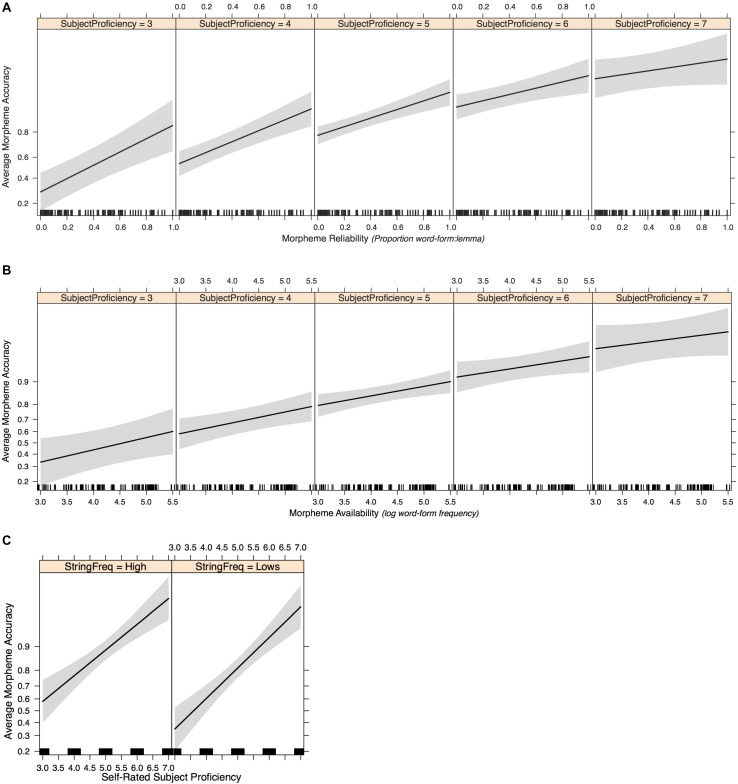
Effect plots of Model 3 fixed-effect interactions: reliability by proficiency **(A)**, availability by proficiency **(B)**, and formulaicity by proficiency **(C)**.

The individualized subject slopes in Figure 7 suggest that participants who have lower proficiency show greater effects of reliability. This interaction seems less marked for effects of availability. To further investigate whether the effects of morpheme reliability, morpheme availability, and formulaicity varied as a function of proficiency, we added the interaction terms into the GLMM (see Table 6 Model 3).

**TABLE 6:** Experiment 2 Model 3 results from the mixed effects model with proficiency and interactions between proficiency and the distribution factors.

		*Fixed effects*				*Random effects*	
						By subject^4^	By item
	
*Parameters*		Estimate	*SE*	*z*	*p*	*SD*	*SD*
Intercept		–6.366	1.939	–3.28	0.001**	0.82	1.00
Morpheme Type^1^	Plural *-s*	–0.888	0.235	–3.78	0.000***		
	Third-person *-s*	0.746	0.231	3.23	0.001**		
	Progressive *-ing*	1.242	0.242	5.14	0.000***		
Morpheme reliability		3.946	1.142	3.46	0.001***		
Morpheme availability^2^		0.591	0.433	1.36	0.172		
Formulaicity (Low)^3^		–1.437	0.614	–2.34	0.019*		
Proficiency		1.234	0.378	3.26	0.001**		
Proficiency*Morpheme reliability		–0.455	0.220	–2.06	0.039*		
Proficiency*Morpheme availability		–0.050	0.085	–0.58	0.559		
Proficiency*Formulaicity		0.165	0.120	1.38	0.168		

^1^Past-tense -ed is the reference level. ^2^Word-form frequency was logarithmically transformed. ^3^High formulaicity was the reference level. Model formula: accuracy ∼ morpheme + proficiency ^∗^ (reliability + availability + formulaicity) + (1| subject) + (1| item). ^4^Random slopes by subject are not included in this model because adding them did not significantly improve model fit [χ^2^(df = 5) = 10.30, p = 0.07]. ***p = 0; **p < 0.001; *p < 0.01; p < 0.1.

Model comparisons using a likelihood ratio test revealed that adding the interaction terms significantly improved model fit [χ^2^(df = 3) = 8.20, *p* = 0.04]. The morpheme type effects were consistent with previous models. The positive effect of morpheme reliability (estimate = 3.946, *SE* = 1.142, *z* = 3.46, *p* = 0.001), formulaicity (estimate = –1.437, *SE* = 0.614, *z* = –2.34, *p* = 0.02), and proficiency (estimate = 1.234, *SE* = 0.378, *z* = 3.26, *p* = 0.001) on morpheme accuracy all remained significant, but the effect of morpheme availability was no longer significant. Notably, there was a significant interaction between proficiency and morpheme reliability (estimate = –0.455, *SE* = 0.220, *z* = –2.06, *p* = 0.04): morpheme reliability effects on production accuracy are greater at lower levels of proficiency (Figure 7A). Proficiency did not interact with the other two distribution factors: the effects of morpheme availability and formulaicity stay the same across different levels of proficiency (Figures 7B,C).

#### Summary of Experiment 2 Results

Experiment 2 replicated the pattern of the rank order difficulty of the four target morphemes: three of the four morphemes conformed to the natural order reported in prior morpheme studies, in that the -*ing* had higher accuracy than the -*ed* and the third-person -*s*. The plural *-s* was again found to be more difficult than what the common order would have predicted. The effects of morpheme availability and reliability were consistent with the results from Experiment 1 and with previous studies. The effects of reliability were greater than those of availability. The interactions with morpheme type confirmed significant reliability effects on Plural *-s*, third person present tense *-s*, and Prog*-ing.* Effects of reliability were greater at lower levels of proficiency; there was no such interaction between availability and proficiency. There were Phrase-Superiority Effects whereby higher frequency four-word strings were associated with increased accuracy of production of morphemes embedded therein. These formulaicity effects were not explicable in terms of sentence length. Participants showed greater accuracy of morpheme provision in sentences they rated as making more sense, though this correlation says nothing about the direction of causation.

## General Discussion

This study investigated ESL learner’s productive morphology in non-obligatory contexts using elicited imitation of sentences containing corpus-sampled morpheme exemplars varying across three probabilistic distribution patterns of item-level features: availability, reliability, and formulaicity. We found that a morpheme is better perceived and more accurately reproduced when (1) it occurs as a word-form that is frequent in usage (i.e., highly available), (2) it is attached to a word that is more consistently conjugated in the form containing this morpheme compared to other forms (i.e., highly reliable), and (3) when the word-form containing it is embedded in a frequent four-word string (i.e., highly formulaic).

### Availability

The facilitation effect of morpheme availability was consistent with the results of prior studies on L1 and L2 acquisition of English morphemes (Larsen-Freeman, 1976; Marchman, 1997; Goldschneider and DeKeyser, 2001; Jia and Fuse, 2007; Räsänen et al., 2016). For example, Marchman (1997) investigated the productive use of English past-tense morphology in children in an elicited-production task and found that errors on regular and irregular verbs (e.g., zero-marking, over-regularization, etc.) were all predicted by item frequency among other factors. Using a similar paradigm, Räsänen et al. (2016) targeted the elicited production of inflectional morphology in L1 Finnish children and found that person/number marked verbs were produced faster and with less errors if they were high-frequency word-forms. Naigles and Hoff-Ginsberg’s (1998) corpus analyses on mother-child conversations collected in naturalistic settings also revealed that the frequency with which verbs appeared in the child-directed speech was significantly predictive of how often and how flexibly the child produced the verbs 10 weeks later. Using production-based measures, Braine et al. (1990) investigate child and adult learning of inflected word-forms of an artificial language and found effects of item and pattern frequency. These include: (1) high frequency morphemes were learned faster and were favored over less frequent ones; (2) item frequency was a significant factor in learning idiosyncratic irregulars; (3) production of a morpheme appropriate to a noun in a generalization test was often affected by whether or not their pairing had been presented previously. Likewise, in adult language acquisition, Finley (2015) trained English speakers on an artificial grammar involving words containing suffixes varying in frequencies. Subsequent testing showed effects upon both memory and generalization: (1) words with high-frequency suffixes were judged to be more acceptable than those with low-frequency suffixes in grammaticality judgment tests where they were to be distinguished from ungrammatical forms, and (2) novel items containing high-frequency suffixes were more likely to be accepted as grammatical compared to those containing low-frequency suffixes. These studies, together with our results of advanced ESL speakers here, all manifest the effects of prior language experience upon processing and cognitive representation. More available forms in usage become entrenched and are more readily perceived and produced by learners (Ellis, 2011).

### Reliability

Usage-based studies of acquisition and processing show that there are a range of different frequencies beyond mere availability that are important in driving the acquisition of linguistic constructions (Ellis, 2012a). The automatic computation that underpins implicit learning does not just tally individual forms or functions, it also automatically learns associations between forms and functions, between forms and other forms, and between forms and contexts (Ellis et al., 2016).

Psychological research into associative learning has long recognized that while frequency of form is important, more so still is contingency of cue-outcome or form-function mapping (Shanks, 1995; MacWhinney, 2001; Ellis, 2006a). Cues with multiple interpretations (i.e., low-contingency) are ambiguous and thus hard to resolve; whereas cue-outcome associations of high-contingency are reliable in their interpretation and readily processed. Consider how, in the learning of the category of birds, while eyes and wings are equally frequently experienced features in the exemplars, it is wings which are distinctive in differentiating birds from other animals. Wings are important features to learning the category of birds because they are reliably associated with class membership while being absent from outsiders. Raw frequency of occurrence is therefore less important than the contingency between cue and interpretation. Reliability of form-function mapping is a driving force of all associative learning, human and animal alike, to the degree that the field of its study has become known as ‘contingency learning.’ This is well recognized in second language acquisition research. For example, Andersen’s (1984) ‘One to One Principle’ of interlanguage construction specifies that an interlanguage system should be constructed in such a way that an intended underlying meaning is expressed with one clear invariant surface form or construction. Contingency learning is also central to the Competition Model, a psycholinguistic theory of language acquisition and sentence processing (MacWhinney, 1987; MacWhinney and Bates, 1989), as well as to other psycholinguistic models of construction learning as the rational learning of form-function contingencies (Ellis, 2006a; Xu and Tenenbaum, 2007).

The competition model focuses upon the various morphological, syntactic, and semantic linguistic cues contained in a sentence – e.g., case marking, word order, and semantic characteristics such as animacy – which people use to interpret the meaning of the sentence. Each cue is probabilistically associated with a particular interpretation, and the cue-weights combine in allowing the learner to choose the interpretation with the highest likelihood. Learners assign cue-weights inductively over their history of experience and usage. Cue-weights differ between languages as different languages use different cues to signal meanings. Thus, second and foreign language learners must learn which cues are important in which languages. To do this, they begin with cues that are more available in the input, after which they come to rely upon cues that are more reliable in their interpretations. Cues that are rare and unreliable are learned late and are relatively weaker, even in adults (MacWhinney, 1997, 2001).

Reliability of association is similarly key in cognitive-linguistic, corpus-based and statistical models of language structure like collostructional analysis (Stefanowitsch and Gries, 2003; Gries and Stefanowitsch, 2004; Gries, 2009). Cognitive linguistic theories of construction grammar focus upon lexical, morphological, and syntactic forms as form-function pairings (Goldberg, 1995, 2006; Hoffmann and Trousdale, 2013). Collostructional analysis focuses more upon form-form reliability in measuring the degree of attraction or repulsion that words exhibit to constructions. It comprises three different methods: (i) collexeme analysis, which measures the degree of attraction/repulsion of a lemma to a slot in one particular construction; (2) distinctive collexeme analysis, which measures the preference of a lemma to one particular construction over another, functionally similar construction; (3) covarying collexeme analysis, which measures the degree of attraction of lemmas in one slot of a construction to lemmas in another slot of the same construction. Collostructional analysis differs from raw frequency counts by providing not only observed co-occurrence frequencies of words and constructions, but also a comparison of the observed frequency to the frequency expected by chance, so as to measure the attraction and repulsion of words and constructions. These measures of association, contingency, and reliability are found to be better predictors of interpretation than are measures of availability (Gries, 2015; Gries and Ellis, 2015; Ellis et al., 2016).

We have already described how in L1 acquisition, the relative frequency of different forms of the same word have been found to predict the usage and error patterns in morpheme acquisition (Bybee, 1985; Hay, 2001; Matthews and Theakston, 2006). There is parallel L2 research showing the importance of reliability. Sugaya and Shirai (2009) described the case study of a native Russian speaker learning Japanese over the course of 10 months. They found that verbs that are more consistently conjugated in a certain common form compared to other possible forms, such as *siru* “come to know” with the imperfective aspect morpheme *-te-i-(ru)*, were produced exclusively in the common form early in the learning trajectory, while this preferential bias was not observed in verbs that do not have a common form. In a recent study on Japanese L1 acquisition, Tatsumi et al. (2018) investigated 3–5-year-olds’ productive use of different forms (simple past tense vs. completive past tense) of verbs in a primed elicited production paradigm, in which the children described actions in line-drawings after hearing the experimenter describing the previous drawing using a verb in the uncommon completive past-tense form. It was found that children’s choice between simple and completive form for each verb reflected the relative frequency of the two forms in corpus data. Although the simple form was generally favored, verbs that have a higher completive past-tense: simple past-tense ratio were more likely to be successfully primed by the experimenter’s use of the completive form, compared to other verbs.

Our present findings further contribute to this growing literature on contingency effects in language processing and production: highly *reliable* morphemes (i.e., exemplars involving lemmas more consistently conjugated in the form containing this morpheme) are more readily acquired and processed.

### Formulaicity and Phrase-Superiority Effects

The results in Experiment 2 demonstrated clear effects of string frequency. There is substantial evidence of chunking and formulaicity effects in first and second language processing and acquisition and in language change (see, for reviews: Ellis, 1996, 2012b, 2017; Wray, 2002; Schmitt, 2004; Siyanova-Chanturia and Pellicer-Sanchez, 2018). For example, Janssen and Barber (2012) demonstrated language learners’ sensitivity to the frequency distributions of multi-word units by having native Spanish speakers produce three types of noun phrases (noun + adjective, noun + noun, determiner + noun + adjective) elicited by line drawings. They found that naming latencies were inversely related to the frequency of the noun phrase but were unrelated to the frequency of the individual words in the phrase. Notably, such formulaicity effects are not restricted to constituents and can span across traditionally defined syntactic boundaries. Tremblay et al. (2011) investigated the processing of non-phrasal sentence segments in a self-paced reading task and found that the frequently occurring “lexical bundles” such as *in the middle of the* were read faster than matched control segments like *in the front of the*. Conklin and Schmitt (2008) embedded such formulaic sequences in short stories and found that they were read faster than matched non-formulaic sequences by both English native speakers and by proficient non-natives. Recent models of sentence processing like the proposals of Christiansen and Chater (2016) on “Chunk-and-Pass” processing have chunking and prediction at their core – we used this model in section “A process analysis of EIT as it relates to morpheme production” to guide our process analysis of EIT.

The fact that the perception and production of a chunk is more affected by the frequency of the chunk than by the frequencies of its component parts suggests that chunks are not fully analyzed into or assembled from their component parts. Bybee (2010) argues that users do not assemble word-forms from their component parts, but rather they store and access them as wholes. Ellis (1996, p. 111) has likewise suggested that formulas might be stored like single “big words.” But there is longstanding debate about whether such formulaic strings are stored as a unit or simply processed preferentially due to context effects and prediction. Likewise in the SLA literature, there are longstanding discussions about whether formulas and idioms are essential parts of the acquisition process or instead are islands of exception, divorced from the language system (Ellis, 2012b; Wulff, 2019).

Our results here show clear effects whereby higher frequency multi-word strings facilitated elicited imitation of the embedded morphemes. We think of these as Phrase-Superiority Effects, the phrasal equivalents of Word-Superiority Effects (WSE, Reicher, 1969; Wheeler, 1970) whereby recognition of a letter is more accurate when it is part of a meaningful word than when it is alone. In WSE experiments, a string of four or five letters is flashed for a few milliseconds onto a screen. Readers are then asked to choose which of two letters had been in the flashed string. For example, if “WORD” had been flashed, a reader might have to decide whether “K” or “D” had been in the final letter position. A WSE arises when subjects choose the correct letter more consistently when letter strings are real words rather than non-words (e.g., “OWRD”) or single letters presented alone (e.g., “___D”). Performance on a forced-choice letter detection task averaged 10% better when the stimuli were four-letter English words than when the stimuli were single letters appearing alone (Wheeler, 1970).

The WSE was a milestone observation in cognitive models of word-recognition and led to the development of the interactive activation model of word recognition (McClelland and Rumelhart, 1981), itself a milestone in the development of connectionist (Parallel Distributed Processing, PDP) models of language. According to this model, when a reader is presented with a word, each letter in parallel either stimulates or inhibits different *feature* detectors (e.g., a curved shape for “C,” horizontal and vertical bars for “T,” etc.). Those feature detectors then stimulate or inhibit different *letter* detectors at a higher level, which finally stimulate or inhibit different *word* detectors at the top-most level. Each activated connection in the large parallel network of connections carries a different weight, and the activation is propagated across levels to give the word detector for “WORD” (in the example above) more activation than any other detector, making “WORD” what the reader eventually recognizes. So far, so bottom-up good. But there are top–down connections too: word detectors pass excitation down to the letter detectors for the letters they contain and inhibit letters they do not; letter detectors pass excitation down to feature detectors for the features they contain and inhibit features they do not. Finally, the model includes inhibitory connections within levels, so that the activation of “WORD” inhibits that of other words, like “WORK,” “WORM,” “LORD,” etc.

The interactive-activation model is a working computational model. It both simulates and explains the WSE as follows: When the target letter is presented within a word, the feature detectors, letter detectors and word detectors are all activated, adding weight to the final recognition of the WORD stimulus, and this in turn sends activation down to its component letters and features. Thus, recognizing the “D” in “WORD” results from activation from both the feature detectors and from the word detectors. However, when recognizing “D” with only the letter presented, there is only the bottom-up activations to the letter detector level. Therefore, perceiving a presented word allows more accurate identification of its component letters, as observed in the WSE.

The WSE demonstrates how frequency and activation at one level of representation (words) may affect the processing and acquisition of linguistic stimuli at another level (letters). Yet ubiquitously, linguistic constructions are inherently nested across various overlapping levels (e.g., morphemes within a word, words within a phrase, phrases within a sentence, all of which can be decomposed into phonemes, etc.). Thus it is likely that there are effects of frequency and contingency across many different grain-sizes of construction, all of which might overlap and interactively activate in intricate ways (Ellis, 2012a; Gries and Ellis, 2015). The Phrase-Superiority effects show how frequency of phrases percolates down to affect the processing of the embedded words and morphemes.

### Between-Morpheme Comparisons

As predicted by the common ESL morpheme acquisition order (Krashen et al., 1977), the *–ing* morpheme was found to be the easiest to acquire by the Chinese participants in our study. This is likely a result from the presence of several facilitating properties such as high level of perceptual saliency, as proposed by Goldschneider and DeKeyser (2001). The morpheme *-ing* is both phonologically salient (because is it a syllabic vowel) and syntactically salient (because its usage is morphosyntactic in nature due to the required co-occurrence of auxiliary *be*). Since auxiliary *be* has been consistently found to be mastered quite well by ESL learners (Krashen et al., 1977), it might have served as an effective cue that have improved the memory for the co-occurring *-ing*. In addition, *-ing* is high in morphophonological regularity and low in semantic complexity, a 1:1 mapping which promotes acquisition.

The past-tense *-ed* morpheme was found to be second easiest to acquire. Besides saliency and regularity, the difference between the processing difficulty of *-ed* vs. *-ing* among Chinese speakers could also be due to typological differences between English and Chinese: namely, English is a “tense-prominent” language whereas Chinese is “aspect-prominent.” It has been reported in the L1 transfer literature that ESL learners who have “aspect-prominent” L1s such as Punjabi use fewer English tense markers such as *-ed* and more aspect markers such as *-ing*, compared to ESL learners whose L1 is the “tense-prominent” Italian (Slobin, 1996). In other words, when exposed to English, Chinese native speakers might habitually pay more attention to aspectually marked verbs than tense-marked verbs.

Interestingly, the plural *-s* morpheme was the lowest in production accuracy in our Chinese speaker sample, which deviates from Krashen et al.’s (1977) common order. This finding is also inconsistent with Tsimpli and Dimitrakopoulou’s (2007) interpretability hypothesis, according to which morphological features related to “interpretable” universal semantic concepts – e.g., plurality – should be easier to acquire than purely grammatical and language-dependent features that lack semantic significance – e.g. the verb agreement on third person singular subjects. One possible explanation why the plural *-s* is difficult for Chinese native speakers lies in the typological differences in how the concept of plurality is expressed between English and Chinese (Jarvis and Pavlenko, 2008; Murakami and Alexopoulou, 2016). In classifier languages like Chinese, plurality (as well as the concept of count/mass distinction) is typically expressed with stand-alone classifiers besides numbers, rather than morphological inflections. Thus, the property (e.g., plurality) of the nouns is entailed by the classifiers that they follow, and not by the nouns themselves. Jiang (2004) argues that Chinese speakers are morphologically insensitive to number information and the count/mass distinction in nouns, especially for those representing abstract concepts, because there is not a classifier that specifically expresses the abstract property of the noun and that is used to count the noun. This also explains why the plural *-s* in the Bottom 10 Reliability group, which consists mostly of abstract nouns, had especially low production accuracy.

Such L1 transfer effects and the resulting deviations from the common order have been demonstrated in previous studies. For example, Luk and Shirai (2009) found that L1 speakers of Japanese, Korean, and Chinese acquire the plural *-s* and articles later than as predicted by the natural order while acquiring the possessive *’s* much earlier. To examine the nature of L1 influence, Murakami and Alexopoulou (2016) conducted a corpus analysis on English morpheme acquisition by ESL learners from seven L1 backgrounds using a database consisting of written exam scripts drawn from the Cambridge Learner Corpus. Usage analyses of six morphemes (articles, past tense *-ed*, plural *-s*, possessive -*’s*, progressive *-ing*, and third person *-s*) revealed significant between-L1 differences, such that L1 type, i.e., whether an equivalent form of the morpheme in English is present or absent in the L1, strongly predicted morpheme accuracy and the order of acquisition. For instance, Japanese L1 speakers tend to score higher on possessive -*’s* and lower on articles than French L1 speakers do, as Japanese lacks the grammatical articles which French has, while French lacks a possessive morpheme which Japanese has. In fact, the lack of L1 equivalence resulted in an accuracy below 90% in almost all morphemes and L1s even among the highly proficient ESL speakers. In addition, the researchers also reported differential influence of L1 type on different morphemes, with articles and progressive *-ing* being the most sensitive to L1 influence, plural *-s* mildly affected, and possessive *-’s* and third-person *-s* the least affected.

Nevertheless, the findings of L1 transfer effects on morpheme acquisition do not deny universal tendencies in the order of morpheme acquisition that are driven by the L2 linguistic input. Corder (1967) noted that what ‘goes on’ in the environment does not equal what actually ‘goes in’ the learner and introduced the concept of ‘intake’ to represent the subset of the available ‘input’ that learners have attended. Second language learners come to the L2 input already “trained” by their prior language experiences to pay different kinds and degrees of attention to patterns in the L2 (Slobin, 1996; Ellis, 2006b). In other words, the between-L1 variance in English morpheme acquisition reflects how learners of different language backgrounds form different ‘focal sets’ through learned selective attention shaped by the nature of their L1, thus transforming different subsets of the L2 ‘input’ into the actual ‘intake’ (Ellis, 2006b). Such attentional bias was demonstrated by Ellis and Sagarra’s (2010) findings that ESL learners whose L1 lacks verb-tense morphology, such as Chinese, were biased to rely more on lexical (e.g., adverbial) cues than on morphological cues to extract temporal information in English. As a result, they experience greater difficulty in acquiring English tense morphemes compared to learners with morphologically rich L1s such as Spanish and Russian. Ellis (2006b) examined moderators of this blocking or “learned attention” bias and proposed that the contingency of form-function mapping in the L2, i.e., reliability, is a significant factor that determines whether input stimuli become the ‘intake,’ further lending support to the reliability effects on ESL morpheme acquisition.

It is important to note that although our design attempted to deny obligatory contexts for using the target morphemes, we achieved this goal with mixed success. The decontextualized nature of EIT denied extra-sentential cues from referential and pragmatic contexts, however, it was more difficult to remove relevant sentence-internal lexical and structural cues. We had greater success removing the cues for plural *-s* than we had for the verbal inflections, and this alone might explain learners’ unexpectedly low performance on this morpheme compared to the others. In contrast, Auxiliary [be] was always provided as a cue for progressive -ing, as needs must, and this may well give progressive *-ing* a processing advantage over the others. Even though we randomly allocated the primacy-denying three-word opening phrases such as *On Saturday morning, Late Wednesday evening, etc*., and these are theoretically tense-neutral, in fact they have tense and modality associations from usage. The *Late Wednesday evening* opening pulls for simple past tense if no auxiliary is provided, and progressive *-ing* if the auxiliary is present. Designating a day of the week and a time of day such as *Late Wednesday evening* implies a more punctual one-time occurrence more than less constrained openings like *On Saturday morning*, and the more specific implication is not always compatible with the Simple Present or Present Progressive. Likewise, the third-person Present *-s* Top 10 reliability set is skewed toward verbs with dummy subjects that only occur in the 3rd-person present in that construction, such as *it concerns, it implies, it consists, it sounds*, and these feel strange in punctual temporal contexts. This range of systemic biases may well have affected accuracy of provision of some morphemes (particularly *-ing* and *-ed*), over others such as (third-person Present *-s*, and Plural -*s* on nouns), and the current research does not allow us to pull these factors apart from the other factors we describe in this section as potential causes of the order of acquisition of different morphemes. These confounds more severely affect cross-morpheme comparisons than they do within-morpheme comparisons of the types discussed in sections “Availability,” “Reliability,” and “Formulaicity and Phrase-Superiority Effects.”

### Subject Proficiency and Interactions With Availability and Reliability

Not surprisingly, proficiency in English was positively associated with accuracy of morpheme provision. Notably, the effects of proficiency did not interact with morpheme availability, suggesting continuity of frequency effects over the learning trajectory. The parallel slopes for effects of frequency at each proficiency stage in Figure 7B, with each proficiency increment increasing the intercept in accuracy, is broadly consistent with usage-based theories of language acquisition which hold that proficiency *is* the cumulative experience of usage frequency. In other words, accumulated language processing leads to the consolidation and entrenchment of linguistic constructions, exemplar by exemplar, and to incremental implicit abstraction of underlying regularities (Bod et al., 2003; Bybee, 2006; MacWhinney and O’Grady, 2014; Schmid, 2017; Schmid, 2020).

On the other hand, proficiency does interact with morpheme reliability. As shown in Figure 7A, reliability effects are much larger early on and gradually decrease as proficiency increases. It seems to be the case that exemplars of high reliability have greater effect at earlier stages of acquisition.

### Why Reliability, Particularly?

Why is reliability of association a more potent determinant than availability? We can make sense of this from the three different perspectives of (1) learning theory, (2) cognitive linguistics, and (3) SLA theory. Indeed, we see their confluence as an important theoretical triangulation where each informs and supports the others.

(1) Associative learning theory demonstrates that contingency of association trumps token frequency (as described in section “Reliability”). In operationalizing reliability here, we focused on how likely it is that a linguistic cue (a morpheme) reliably co-occurs with another (a lemma). But morphemes and lemmas go beyond being mere forms, they are linguistic constructions with particular functions and meanings: they are symbolic.

(2) Cognitive linguistic theories of construction grammar viewlexical, morphological, and syntactic forms as symbolic form-functionpairings and hold that we learn language from usage. Collostructional analysis focuses as much upon form-form reliability in measuring the degree of attraction or repulsion that words exhibit to constructions. When learners are processing usage, they are tallying the associations between forms, between interpretations, and between forms and their interpretations. Verbs have interpretations and so do morphemes and these can vary in their form-function reliability. Verbs and morphemes can be more or less reliably associated (form-form reliability). The matrix of association goes beyond mere forms; in full it involves:


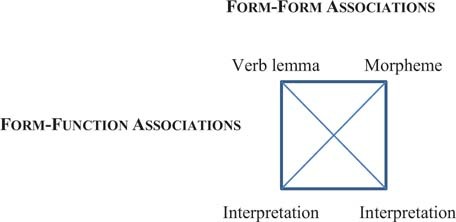


(3) Functional theories of SLA emphasize the interplay of form and meaning in acquisition. One much-researched example for morphology is the Aspect Hypothesis (AH) (Andersen and Shirai, 1996; Bardovi-Harlig, 2000; for a state-of-the-scholarship review of the last 20 years of research, see Bardovi-Harlig and Comajoan-Colomé, 2020). The AH builds on three main constructs: tense, grammatical aspect, and lexical aspect. Tense establishes the location of an event (or situation) in time with respect to the moment of speech or some other reference point. Grammatical aspect allows for “ways of viewing the internal temporal constituency of a situation” (Comrie, 1976, p. 3). For instance, in English, a contrast in grammatical aspect is found between simple past “John walked” and past progressive “John was walking.” In contrast, lexical aspect refers to semantic differences in verbs and their arguments (Dowty, 1991), such as whether a predicate has inherent duration [e.g., “walk,” “sleep,” and “kid (v.)”], or is punctual (e.g., “recognize,” “broke,” and “sigh”), or has elements of both duration and culmination (e.g., “walk a mile” and “paint a picture”). The AH predicts that “second language learners will initially be influenced by the inherent semantic aspect of verbs or predicates” (Andersen and Shirai, 1996, p. 533). “In its simplest form, the AH for SLA predicts that in the initial stages of the acquisition of tense-aspect morphology by adults, the acquisition of past morphology will be influenced by lexical aspectual categories. Namely, verbal morphology will be attracted to and will occur with predicates with similar semantics. Perfective past will occur with telic predicates (i.e., those with inherent endpoints), imperfective will occur with unbounded predicates, and progressive will occur with ongoing activities” (Bardovi-Harlig and Comajoan-Colomé, 2020, p. 3). Bardovi-Harlig and Comajoan-Colomé conclude from their review of perhaps thirty different studies that the AH accurately predicts the adult L2 acquisition of past morphology in a number of languages.

Our research in this article has demonstrated effects of distributional learning – particularly the privileged processing of reliably associated lemma-morpheme pairings (**form-form reliability**). The supplementary question that naturally follows is to wonder why language is distributed this way. Cognitive linguistics more generally, and the AH in particular, suggest that for the case of tense-aspect morphology, there are semantic and functional motivations. Likewise, for noun number, we suspect that inherent number, pluralia tantum, and prototypically plural count nouns might lead the way. These are effects of **form-function reliability**. Form-form and form-function associations interact in various complex and adaptive ways in usage, and a speaker’s language system reflects the history of their processing these associations. There is good reason and plenty of scope to study a broad range of morphology in this way.

## Limitations

There are various limitations to this study. (1) It is relatively small in size in terms of its sample of participants and its sample of stimuli^11^. (2) We are also concerned just how representative the corpus is for our participants. We drew our stimuli from the largest existing corpus of American English, COCA (Davies, 2008), assuming it to truly represent the English exposure and usage of the non-native English-speaking participants living in the US. This assumption can be problematic as the English used by the general population as the L1 might well vary from that used as an L2 in pedagogical settings. Despite our best efforts, participants might not be familiar with all the vocabulary in the sentences, and particularly, with the target words. If they did not know the target word, it seems likely that the whole word would be omitted in the response. Although we tried to mitigate the problem by only scoring the morpheme provision when the correct lemma is present, it still unavoidably resulted in an unequal number of missing cases across the ARD groups, with the highest exclusion rate in the bottom frequency and proportion group. (3) In creating the stimuli for Experiment 2, in order to control a range of important potential other factors, in making the low-frequency sentence strings, we replaced (high-frequency and shorter pronouns) with (lower frequency and longer proper nouns). This systematic confound introduces uncertainly into whether any effects of this manipulation result from sentence string frequency, length, or pronoun vs. noun. We were at least able to show that string frequency was a much more important effect than length. Nevertheless, if possible, it would be good to avoid such confounds in future research. (4) Our design attempted to provide non-obligatory contexts for using the target morphemes, but we achieved this goal with mixed success. The decontextualized nature of EIT denied extra-sentential cues from referential and pragmatic contexts, however, it was more difficult to remove relevant sentence-internal lexical and structural cues. For example, Auxiliary [be] was always provided as a cue for progressive -ing. This, and the range of other factors detailed at the end of section “Between-Morpheme Comparisons,” introduce a range of factors that deny simple identification of the causes of between-morpheme differences. (5) Our quest for control and the matching of the stimuli in terms of several dimensions of corpus metrics resulted in stimulus sentences that are somewhat uneven in their approximation of naturally occurring English. (6) Our chosen experimental paradigm, the Elicited Imitation Test, targets decontextualized language repetition rather than situations of rich, meaningful communication where there is clearly more scope for the importance of word meanings and other form-function associations. (7) Adapting the EIT for typed rather than spoken responding potentially allows more influence of considered explicit processing in the written responses, although the window for these influences comes after online listening, which we believe to be the rate-limiting step which maximizes demands for implicit or automatized processing. However, further research involving spoken responding would be useful for triangulation.

For future research, we encourage the analysis of large learner corpora (of the type exemplified by, e.g., Murakami and Alexopoulou, 2016) in order to broaden the investigation to many more learners, large amounts of more communicative natural language, a wider range of morphology, and a focus upon participant effects (including L1 transfer, longitudinal development, proficiency, etc.). Widening the range of languages studied is also a priority.

## Conclusion

We investigated usage-based effects of availability, reliability, and formulaicity in ESL acquisition of inflectional morphemes: -*ed, -ing*, and 3rd-person *-s* on verbs, and plural -*s* on nouns and showed using EIT that morphemes were more easily processed when they were (1) available (occurring in frequent word-forms), (2) reliable (occurring in lemmas consistently conjugated in this form), and (3) formulaic (embedded in high- vs. low-frequency phrases). Such conclusions support cognitive theories of the statistical symbolic learning of morphology. Language acquisition reflects the distributional properties of the linguistic input at multiple grain-sizes.

## Data Availability Statement

The raw data supporting the conclusions of this article will be made available by the authors, without undue reservation.

## Ethics Statement

The studies involving human participants were reviewed and approved by University of Michigan IRB Health Sciences and Behavioral Sciences (HSBS). The patients/participants provided their written informed consent to participate in this study.

## Author Contributions

Both authors listed have made a substantial, direct, and intellectual contribution to the work and approved it for publication.

## Conflict of Interest

The authors declare that the research was conducted in the absence of any commercial or financial relationships that could be construed as a potential conflict of interest.

## Footnotes

1 Friends share mappings from stem to tense (e.g. *walk-walked, talk-talked*) and pull together; enemies use different mappings (e.g., *throw-threw*) and pull apart (Marchman et al., 1999).

2 There are alternative operationalizations available for formulaicity involving mutual information or other measures of conditional probability rather than string frequency, and these various measures can affect acquisition and processing in different ways (e.g., Ellis, 2012b). Here we adopt simple string frequency because it is the most basic and widely used metric for formulaic language, multi-word expressions, and lexical bundles (Wray, 2002; Biber, 2004; Gries and Ellis, 2015).

3 Searching for this frame in COCA returns more than 1600 verb types, in decreasing order: *do, make, get, see, keep, take, put, say*, … following a Zipfian frequency distribution).

4 A visiting student who had just arrived in the US for the first time was included in the study because of her demonstrable fluency in English and the fact that she had been receiving part-time English-immersed education in China.

5 https://corpus.byu.edu/coca/

6 More information regarding the syntax can be found at the BYU corpus portal (corpus.byu.edu) and from the “insert POS” tab in search page of COCA (https://corpus-byu-edu.proxy.lib.umich.edu/coca/)

7 https://www.audacityteam.org/

8 Most of the Experiment 1 sentences remained exactly the same except 24 sentences. The four-word string of 13 sentences were changed to include at least one animate pronoun (e.g., changing “*it seemed to be*” to “*he seemed to be*”). Eleven sentences were slightly shortened so that both the high formulaicity and the low formulaicity versions were between 14 and 17 syllables long.

9 All the person names were selected from the top 100 female and top 100 male names used in the United States over the last 100 years according to the Social Security census results (available at https://www.ssa.gov/oact/babynames/decades/century.html).

10 Like Experiment 1, in order to determine which subject-level random slopes to include, we ran each of the models in Experiment 2 both with and without subject random slopes and compare the fit of the two models using a likelihood ratio test. We included the subject random slope for a fixed-effect predictor only when this addition significantly improves model fit. Otherwise, we report the model without the subject random slope (i.e., the one with random intercept only).

11 Although, in defense, we point out that there is replication research here between Experiments 1 and 2, and that the stimuli were carefully chosen from a corpus of over 560 million words to lie at the interesting extremes to afford maximal power.
