# Supplementary figures and images for: Language Usage and Second Language Morphosyntax: Effects of Availability, Reliability, and Formulaicity
Source: Front Psychol. 2021 Apr 29;12:582259. doi: 10.3389/fpsyg.2021.582259 (PMC8116661; doi:10.3389/fpsyg.2021.582259)

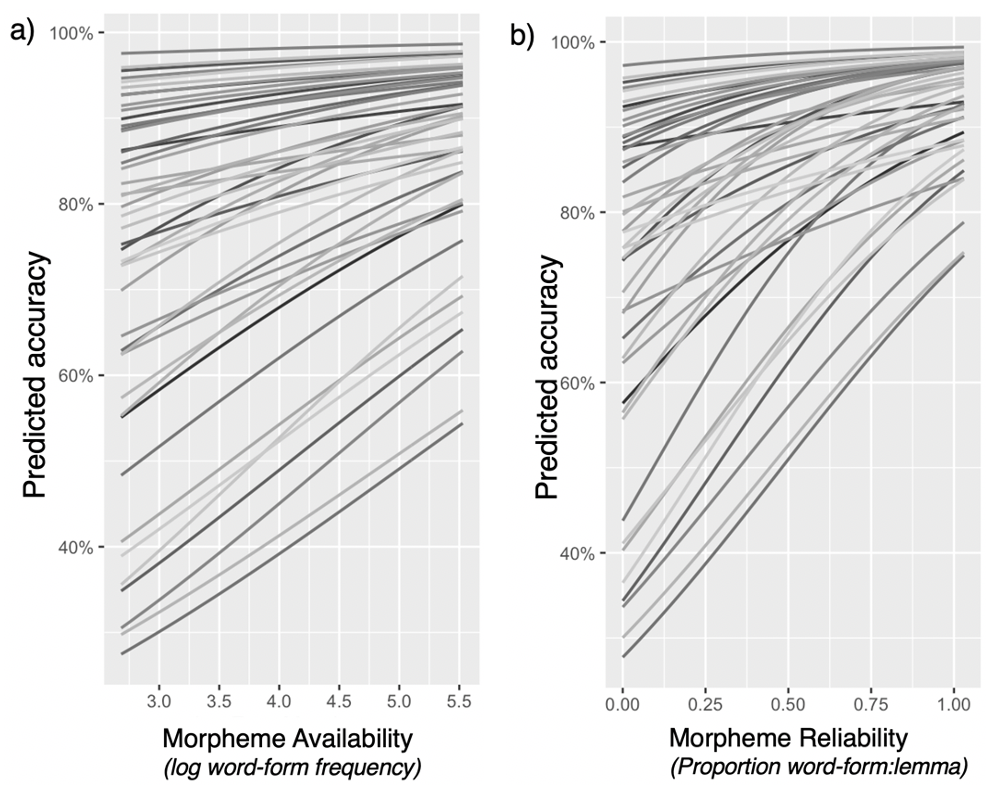

Supplement: Supplementary file 1 [file Image_1.JPEG]
