# Supplementary material for: Language Usage and Second Language Morphosyntax: Effects of Availability, Reliability, and Formulaicity
Source: Front Psychol. 2021 Apr 29;12:582259. doi: 10.3389/fpsyg.2021.582259 (PMC8116661; doi:10.3389/fpsyg.2021.582259)

Supplementary Material

# Supplementary Data and Analyses

## Demographic information and language experience of participants in Experiment 1.

|  |  | Mean | SD | Min | Max |
| --- | --- | --- | --- | --- | --- |
|  | Length of residence (mo) | 31.33 | 21.69 | 6 | 84 |
|  | Onset of learning (age) | 7.05 | 2.68 | 2 | 14 |
|  | Onset of arrival (age) | 18.27 | 4.18 | 10 | 28 |
| TOEFL Score | Total (/120) | 103.21 | 5.86 | 93 | 114 |
|  | Reading (/30) | 27.91 | 2.30 | 23 | 30 |
|  | Writing (/30) | 27.30 | 1.49 | 25 | 29 |
|  | Listening (/30) | 26.67 | 2.92 | 21 | 30 |
|  | Speaking (/30) | 23.40 | 1.84 | 20 | 26 |
| Amount of English exposure (hrs/wk) | Total | 45.41 | 30.96 | 10 | 145 |
|  | Reading | 9.16 | 7.96 | 0 | 30 |
|  | Writing | 6.80 | 4.32 | 0 | 15 |
|  | Listening | 17.36 | 18.11 | 2 | 80 |
|  | Speaking | 12.09 | 11.87 | 1 | 40 |
| Self-rated Proficiency (1-7) | General | 5.18 | 0.96 | 3 | 7 |
|  | Reading | 5.27 | 0.77 | 4 | 6 |
|  | Writing | 4.95 | 0.95 | 3 | 7 |
|  | Listening | 5.09 | 1.15 | 3 | 7 |
|  | Speaking | 4.77 | 1.31 | 3 | 7 |

## The initial steps in building up the GLMM analyses of Experiment 1

### Morpheme Type alone

We started with a mixed-effects logit model with morpheme type as the only fixed-effect predictor, and random intercepts for subjects and items. Production accuracy was specified as a binary outcome. Levels of the categorical predictor morpheme type was reference coded in R by default, with a 0 assigned to the Past-tense *-ed* as the reference level, and 1 assigned to the other three morphemes. Results indicated that morpheme type was significantly related to production accuracy. Specifically, *-ing* had significantly higher accuracy than *-ed* (estimate = 1.028, SE = . 472, z = 2.18, p < .05); the Plural *-s* had significantly lower accuracy than *-ed* (estimate = -.877, SE = .427, z = -2.05, p < .05); the 3^rd^-person *-s* had marginally lower accuracy than *-ed* (estimate = -.725, SE = .425, z = -1.71, *p =* .088). The Analysis of Deviance using Type III Wald chi*-*square tests indicated that morpheme type was a significant predictor to be included in the model (χ^2^(df = 3) = 21.37, *p =* .000). Adding random slope for subjects for morpheme type does not improve model fit (χ^2^(df = 9) = 9.502, p = .392), and thus it is left out of subsequent analyses.

### Morpheme Type + Morpheme Reliability

To examine the unique contribution of morpheme reliability, the calculated reliability was added to the previous model as a second fixed-effect predictor in addition to morpheme type, with all the analyses remain the same. The fixed effects of morpheme type were mostly consistent with the results from the previous model: *-ing* had significantly higher accuracy than *-ed* (estimate = 1.104, SE = . 435, z = 2.54, *p =* .01); the Plural *-s* had significantly lower accuracy than *-ed* (estimate = -1.299, SE = .404, z = -3.21, *p =* .001). The difference between the 3^rd^ person present tense *-s* and *-ed* was no longer significant (estimate = -.453, SE = .390, z = -1.16, *p =* .245). More notably, reliability emerged as a significant positive predictor of accuracy (estimate = 2.150, SE = .513, z = 4.20, *p =* .000). Analysis of Deviance using Type III Wald chi*-s*quare tests showed that both morpheme type and reliability were significant predictors of accuracy (morpheme type: χ^2^(df = 3) = 32.02, *p =* .000; reliability: χ^2^(df = 1) = 17.60, *p =* .000), confirming the unique contribution of morpheme reliability, with morpheme type taken into account. Adding random slope for subject for morpheme reliability does not improve model fit (χ2 (df = 2) = .478, p = .787), and thus it is left out of subsequent analyses.

### Morpheme Type + Morpheme Availability

To examine the unique contribution of morpheme availability, we ran the same mixed-effects logit model with morpheme type and the log frequency of the word-form as two fixed-effect predictors, with all the analyses remained the same. The frequency was logarithmically transformed to compress the frequency range and minimize nonlinearity (see Seidenberg & McClelland, 1989 for discussion on frequency range compression). Again, the fixed effects of morpheme type were largely consistent with results from previous models: *-ing* had higher accuracy than *-ed*, although the effect is less significant (estimate = .847, SE = .442, z = 1.92, *p =* .055); the Plural *-s* had significantly lower accuracy than *-ed* (estimate = -1.210, SE = .411, z = -2.94, p < .005). The difference between the 3^rd^ person present tense *-s* and *-ed* was not significant (estimate = -.623, SE = .396, z = -1.57, *p =* .116). The positive effect of log word-form frequency was highly significant (estimate = .661, SE = 0.196, z= 3.37, *p =* .000). Analysis of Deviance using Type III Wald chi*-*square tests showed that both morpheme type and word-form frequency were significant predictors of accuracy (morpheme type: χ^2^(df = 3) = 25.687, *p =* .000; morpheme availability (log word-form frequency): χ^2^(df = 1) = 11.352, *p =* .000), confirming the unique contribution of the availability of the morpheme, with the morpheme type taken into account. Adding random slope for subject for morpheme reliability does not improve model fit (χ2 (df = 2) = .447, p = .8), and thus it is left out of subsequent analyses.

## Demographic information and language experience of participants in Experiment 2.

|  |  | Mean | SD | Min | Max |
| --- | --- | --- | --- | --- | --- |
|  | Length of residence (mo) | 44.28 | 48.15 | 0.5 | 192 |
|  | Onset of learning (age) | 7.96 | 3.03 | 2 | 14 |
|  | Onset of arrival (age) | 15.30 | 6.91 | 3 | 37 |
| TOEFL Score | Total (/120) | 105.48 | 5.01 | 96 | 114 |
|  | Reading (/30) | 27.91 | 2.88 | 16 | 30 |
|  | Writing (/30) | 26.68 | 2.15 | 22 | 30 |
|  | Listening (/30) | 27.00 | 2.29 | 20 | 30 |
|  | Speaking (/30) | 24.05 | 2.66 | 21 | 30 |
| Amount of English exposure (hrs/wk) | Total | 52.73 | 76.53 | 5 | 392 |
|  | Reading | 10.74 | 13.00 | 0 | 84 |
|  | Writing | 9.51 | 13.54 | 0 | 76 |
|  | Listening | 18.33 | 35.05 | 1 | 168 |
|  | Speaking | 14.14 | 28.81 | 0 | 168 |
| Self-rated Proficiency (1-7) | General | 5.04 | 0.88 | 3 | 7 |
|  | Reading | 5.29 | 0.99 | 3 | 7 |
|  | Writing | 4.58 | 1.06 | 2 | 6 |
|  | Listening | 5.16 | 1.21 | 6 | 7 |
|  | Speaking | 4.80 | 1.39 | 2 | 7 |

## Experiment 2 results from the mixed effects model including fixed effects of morpheme type, morpheme reliability (proportion), and morpheme availability (log word-form frequency) and random effects of subject and item.

|  | | | *Fixed effects* | | | |  | *Random effects* | |
| --- | --- | --- | --- | --- | --- | --- | --- | --- | --- |
|  | |  |  |  |  |  |  | By Subject | By Item |
| *Parameters* | | | Estimate | SE | z | p |  | SD | SD |
| Intercept | | | -0.346 | 0.575 | -0.60 | 0.55 |  | 1.740 | 1.069 |
| Morpheme Type^1^ | Plural *-s* | | -0.881 | 0.247 | -3.57 | 0.000 | *** |  |  |
|  | 3rd-person *-s* | | 0.752 | 0.241 | 3.11 | 0.002 | ** |  |  |
|  | Progressive *-ing* | | 1.255 | 0.252 | 4.99 | 0.000 | *** |  |  |
| Morpheme Reliability | | | 1.720 | 0.350 | 4.91 | 0.000 | *** | 0.822 |  |
| Morpheme Availability^2^ | | | 0.319 | 0.126 | 2.52 | 0.012 | * | 0.171 |  |

*Note.* ^1^ The Past-tense *-ed* is the reference level; ^2^ Word-form frequency was logarithmically transformed. Model formula: accuracy ~ morpheme + reliability + availability + (1+reliability+availability|subject)+(1|item).

# Supplementary Experimental Materials

## The full list of Expt. 1 and Expt. 2 stimulus words with their lemma frequency, word-form frequency, and the calculated reliability

Please see the Excel file named “Full Stimuli List.xlsx”

## Language Background Questionnaire

Please see the PDF file named “Language background questionnaire.pdf”

# Supplementary Figures

## Predicted effects of availability (a) and reliability (b) in each subject in Experiment 2


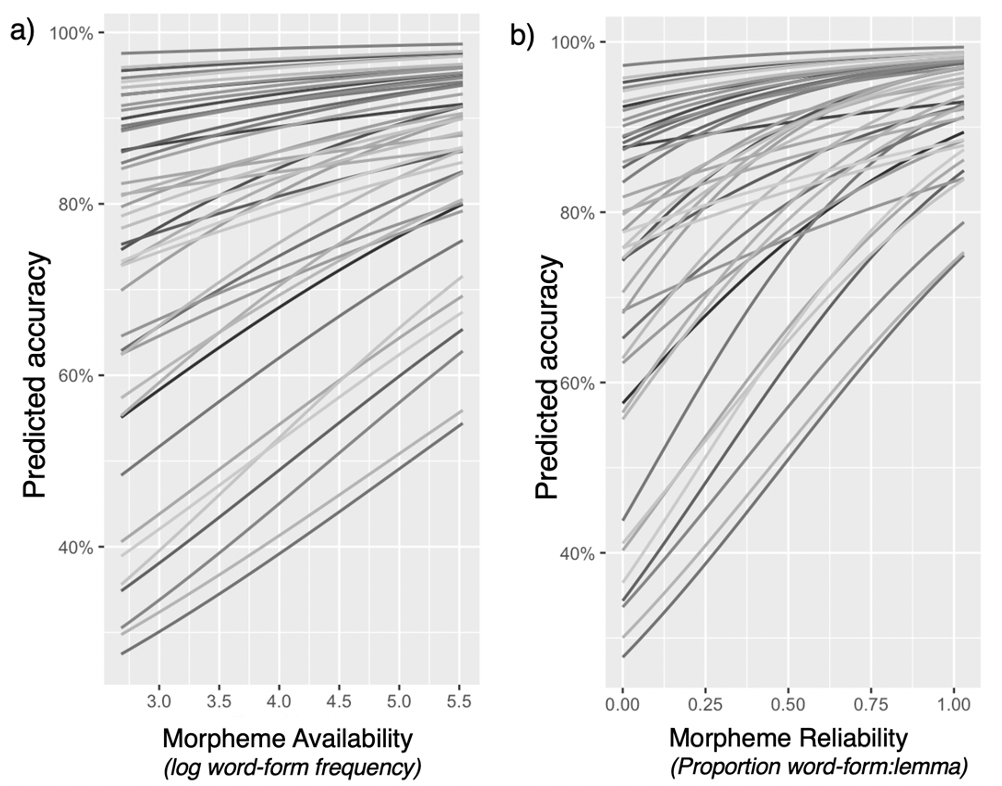

Supplement: Supplementary file 2 [file Data_Sheet_1.docx]
