# Supplementary material for: Language Usage and Second Language Morphosyntax: Effects of Availability, Reliability, and Formulaicity
Source: Front Psychol. 2021 Apr 29;12:582259. doi: 10.3389/fpsyg.2021.582259 (PMC8116661; doi:10.3389/fpsyg.2021.582259)
Supplement: Supplementary file 4 [file Data_Sheet_1.PDF]

Subject ID: C_____

Language Background Questionnaire

1. Gender: Male ☐ Female ☐
2. Age: ______
3. Native language(s): _______________
4. Other language(s) spoken at home as a child: _______________
5. Have you learned any foreign languages other than English? ☐Yes ☐No
   If yes, please specify all information including 1) which language, 2) how long, and 3) in what context(s)
   I have learned _______________ for ___ year(s) and ___month(s). Context: ___________________
   I have learned _______________ for ___ year(s) and ___month(s). Context: ___________________
   [Example] I have learned French for 1 years and 6 months as a part of my coursework in high school.
   I have learned German for 2 years because my family moved to Germany during that time.
6. **At what age** did you **begin studying English**? ______
7. How long **in total** have you been studying in English-speaking countries (including previous education)?
   For ______ years ______ months.
8. **At what age** were you **first exposed** to English communication environment? ______
   For how long? ______
9. TOEFL Score (if you remember)
   Total score: ______ (Reading: ______ Writing: ______ Listening: ______ Speaking: ______)
   Test Date: _____________________ (e.g., summer 2007)
10. How much time do you usually spend using English from outside of the classroom?

Language Skills	Description	Use of Time (Weekly)
Reading	Newspaper (including online or internet news), magazines, books (including textbooks), academic journals, and any other types of reading activities in English	(about _____ hours) per week
Writing	Homework assignment, essay, diary, texting/messaging in English, and any other types of writing activities in English	(about _____ hours) per week
Listening	Pop music (in English), lectures, TV, radio, and any other types of listening activities in English	(about _____ hours) per week
Speaking	Conversing with friends who are native speakers of English, in-class discussions in English, and any other types of speaking activities	(about _____ hours) per week

11. On a scale of 1-7, please self-rate your English proficiency in general and in terms of different skills (with higher scores corresponding to higher proficiency):

General proficiency:	1	2	3	4	5	6	7
Reading:	1	2	3	4	5	6	7
Writing:	1	2	3	4	5	6	7
Listening:	1	2	3	4	5	6	7
Speaking:	1	2	3	4	5	6	7

Your privacy will be protected. Thank you for your participation.
